# Overlapping and Distinct Mechanisms of Effective Neoantigen Cancer Vaccines and Immune Checkpoint Therapy

**DOI:** 10.1101/2023.12.20.570816

**Published:** 2023-12-22

**Authors:** Sunita Keshari, Alexander S. Shavkunov, Qi Miao, Akata Saha, Charmelle D. Williams, Anna M. Highsmith, Josué E. Pineda, Elise Alspach, Kenneth H. Hu, Kristen E. Pauken, Ken Chen, Matthew M. Gubin

**Affiliations:** 1. Department of Immunology, The University of Texas MD Anderson Cancer Center, Houston, TX, USA; 2. Department of Bioinformatics and Computational Biology, The University of Texas MD Anderson Cancer Center, Houston, TX, USA; 3. Department of Molecular Microbiology and Immunology, Saint Louis University School of Medicine, St. Louis, MO, USA; 4. The Parker Institute for Cancer Immunotherapy, The University of Texas MD Anderson Cancer Center, Houston, TX, USA; 5. The James P. Allison Institute, The University of Texas MD Anderson Cancer Center, Houston, TX, USA

## Abstract

The goal of therapeutic cancer vaccines and immune checkpoint therapy (ICT) is to eliminate cancer by expanding and/or sustaining intratumoral T cells with enhanced anti-tumor capabilities. However, whether therapeutic cancer vaccination and ICT achieve enhanced anti-tumor immunity by distinct or somewhat overlapping immunological mechanisms remains unclear. Considering increasing interest in combining these two types of treatment to improve efficacy rates, a better understanding of how these treatments are similar and different is needed. Here, we compared effective therapeutic tumor-specific mutant neoantigen (NeoAg) cancer vaccines with anti-PD-1, anti-CTLA-4, or anti-CTLA-4 plus anti-PD-1 combination ICT in preclinical models. We found that both NeoAg vaccines and anti-CTLA-4 and/or anti-PD-1 ICT induced robust expansion of intratumoral NeoAg-specific CD8 T cells, though the degree of expansion and acquisition of effector activity was more substantial following NeoAg vaccine compared to ICT. Further, we found that NeoAg vaccines are particularly adept at inducing proliferating and stem-like NeoAg-specific CD8 T cells. Additionally, anti-CTLA-4 notably induced ICOS^+^ Th1-like CD4 T cells expressing the transcription factor Bhlhe40 and, interestingly, when combined with anti-PD-1 a small subset of Th2-like CD4 T cells was observed. Conversely, we observed a more divergent effect on certain subsets of intratumoral macrophages induced by NeoAg vaccines as compared to ICT. Although effective NeoAg vaccines or ICT expanded M1-like iNOS^+^ macrophages, NeoAg vaccines expanded rather than suppressed (as observed with ICT) distinct subpopulations of M2-like CX3CR1^+^ CD206^+^ macrophages, associated with the poly I:C adjuvant used in the vaccine. Considering the similarities and difference we identified in how NeoAg vaccines versus ICT reshaped the TME, we hypothesized that combining ICT with NeoAg vaccines would expand the therapeutic window for efficacy in these preclinical models. Indeed, we found the combination NeoAg vaccine plus ICT induced superior anti-tumor control compared to either therapy in isolation, highlighting the utility of combining these modalities to eliminate cancer.

## INTRODUCTION:

For cancer immunotherapies such as ICT, T cell recognition of tumor antigens is critical for efficacy^1^. In contrast to aberrantly expressed non-mutant antigens, tumor-specific neoantigens (NeoAgs) formed from somatic alterations in cancer cells are not subject to immune tolerance and are exclusively expressed in cancer cells, making them favorable cancer vaccine targets^2-4^. Significant progress has been made in the field of NeoAg cancer vaccine development, showing promise in early-phase clinical trials^5-11^. Despite this progress, many fundamental questions regarding NeoAg vaccines remain unclear^5,6,10,12^, including how to best combine therapeutic vaccines with other T cell-directed therapeutic modalities including ICT to promote optimal outcomes in cancer patients. A more refined understanding of how NeoAg vaccines impact the immune tumor microenvironment (TME) in comparison to other immunotherapies like anti-PD-1 and anti-CTLA-4 ICT can inform rational use of NeoAg vaccines and combinatorial immunotherapies.

To address this, we developed preclinical models to interrogate potential synergies between the mechanisms of action of NeoAg cancer vaccines and ICT. We previously used immunogenomic/mass spectrometry approaches to identify NeoAgs and subsequently demonstrated that therapeutic NeoAg cancer vaccines could provoke tumor rejection in methylcholanthrene (MCA)-induced sarcoma models^4,13^. Others have used similar approaches to identify immunogenic NeoAgs^4,6,7,14-16^. We further showed that NeoAgs are major targets of T cells reactivated by ICT and that anti-PD-1 and anti-CTLA-4 administered either alone or in combination induces changes in both CD4 and CD8 T cells within the TME^13,17-20^, consistent with findings from others^21,22^. While both conventional CD4 and CD8 T cells drive immunotherapeutic responses to cancer, cytotoxic CD8 T cells are typically the most potent direct inducers of tumor cell death. In both cancer patients and preclinical models, intratumoral CD8 T cells that express activation markers including inhibitory receptors such as PD-1, LAG-3, and TIM-3 often exist in a terminally differentiated state and may display a range of functional capabilities from short-lived CD8 T effector cells with potent cytotoxicity and cytokine production to dysfunctional or exhausted CD8 T cells that exist in a state of limited or restrained functional capabilities^23^. These dysfunctional or exhausted CD8 T cells also exist on a spectrum of intermediate to terminally dysfunctional/exhausted with progression to terminally dysfunctional/exhausted characterized by high, sustained expression of inhibitory receptors, reduced function, and unique transcriptional and epigenetic profiles, differentiating them from memory T cells and T cells displaying stem-like properties (often referred to as progenitor/precursor exhausted CD8 T cells). These distinct states are driven by key transcription factors, including TCF-1, which promotes stemness or memory-like attributes^24^, and TOX, which plays a crucial role in establishing terminal dysfunction/exhaustion^25-27^. Although TOX is induced in an NFAT-dependent manner upon T cell activation, chronic antigen exposure and/or signals within the TME promote maintenance of NFAT-independent TOX expression and establishment of a fixed epigenetic landscape in terminal dysfunctional/exhausted CD8 T cells^28^. The increased presence of PD-1^hi^ TOX^+^ TCF-1^−^ CD8 T cells in tumor biopsies correlates with a poorer prognosis in patients treated with ICT and these cells likely do not drastically gain effector function following PD-1/PD-L1 blockade. Instead, stem-like PD-1^+^ Tim-3^−^ TCF-1^+^ CD8 T cells within tumors and lymph nodes expand and differentiate into PD-1^+^ Tim-3^+^ CD8 T effector-like cells in response to anti-PD-1/PD-L1 ICT^29,30^.

While T cells are the major target of NeoAg vaccines and ICT, myeloid cells are a critical component of the TME^31^. Macrophages are amongst the most abundant myeloid cell population within tumors and may comprise both embryonically-derived tissue-resident macrophages and monocyte-derived macrophages^32-35^. Although macrophages can exhibit anti-tumor effects, macrophages often promote tumor growth. We previously observed major complexity in the ICT-induced changes occurring in the intratumoral macrophage compartment^18-20^. These included remodeling from M2-like CX3CR1^+^ CD206^+^ macrophages in progressively growing tumors to M1-like iNOS^+^ macrophages in tumors that go on to reject in response to ICT. Further, blockade of TREM2 expressed on macrophages using Fc-mutated anti-TREM2 monoclonal antibody (mAb) induced a decline in CX3CR1^+^ CD206^+^ macrophages and induced iNOS^+^ macrophages and subsets of macrophages expressing immunostimulatory molecules, with anti-TREM2 dampening tumor growth and augmenting anti-PD-1 efficacy^36^. While tumor immune cell compositions clearly play a major role in response to immunotherapy^33,37^, the heterogeneity and dynamics of immune infiltrates in response to immunotherapies such as NeoAg cancer vaccines is not thoroughly characterized. Further, although much progress has been made towards defining the mechanisms behind ICT efficacy, our understanding is still incomplete and direct comparisons between cancer vaccines and different ICTs used alone or in combination are largely lacking.

Here, we systematically compared different immunotherapies that lead to tumor rejection, including NeoAg cancer vaccines, anti-PD-1, anti-CTLA-4, and anti-PD-1 + anti-CTLA-4 ICT using mouse melanoma models expressing defined NeoAgs. NeoAg vaccines induced robust expansion of polyfunctional NeoAg-specific CD8 T cells, including proliferating and stem-like CD8 T cells. Anti-CTLA-4 and/or anti-PD-1 ICT increased the frequency and effector function of intratumoral NeoAg-specific CD8 T cells, with anti-CTLA-4 containing treatments also dramatically altering the CD4 T cell compartment. Both NeoAg vaccines and ICT resulted in an expansion of M1-like iNOS^+^ macrophages. Whereas ICT reduced the frequency of intratumoral CX3CR1^+^ CD206^+^ M2-like macrophages, NeoAg vaccine treated mice instead displayed a higher frequency of CX3CR1^+^ CD206^+^ macrophages. Thus, effective NeoAg cancer vaccines reshape the TME, leading to overlapping as well as distinct alterations when compared to different ICT treatments. To investigate whether the unique impacts of NeoAg vaccines and ICT combine for enhanced tumor control, we tested the efficacy of NeoAg vaccination in combination with either anti-CTLA-4 or anti-PD-1 and found that the window of therapeutic efficacy was extended by combination treatments, further supporting the rationale of combining NeoAg vaccines with ICT.

## RESULTS:

### NeoAg vaccines and ICT induce T cell-dependent long-term tumor protection

**Figure 1.**
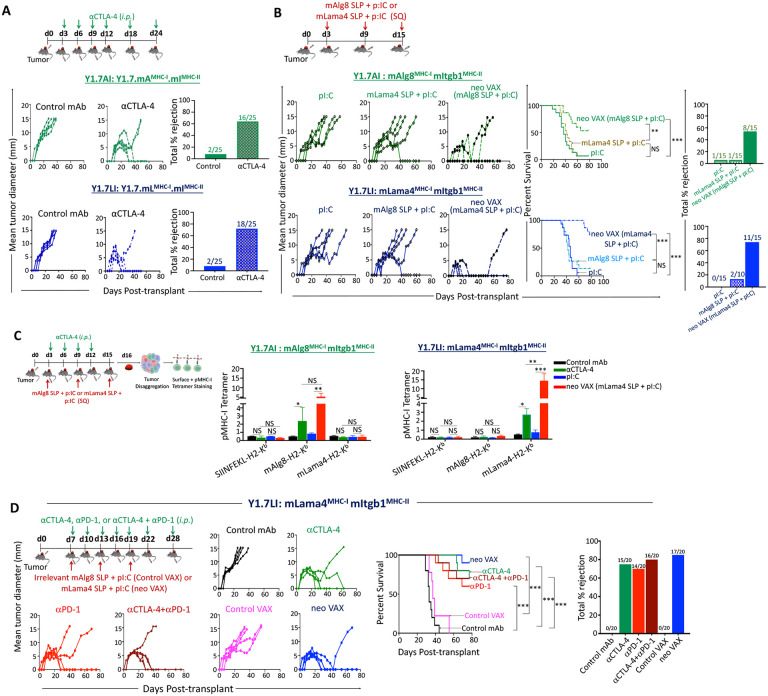
Therapeutic NeoAg Vaccines or ICT Inhibit Tumor Growth in Mice Bearing NeoAg-Expressing *Braf*^*V600E*^ *Pten*^−/−^ *Cdkn2a*^−/−^ Melanoma. **(A)** Tumor growth and percent tumor rejection in WT C57BL/6J mice transplanted with Y1.7 mA^MHC-I^.mI^MHC-II^ (Y1.7AI) and Y1.7 mL^MHC-I^.mI^MHC-II^ (Y1.7LI) melanoma cells and treated with control mAb or anti-CTLA-4 immune checkpoint therapy (ICT) starting on d. 3 post tumor-transplant, and subsequently on d. 6, 9, 12, 18, 24. (**B**) Tumor growth, cumulative mouse survival, and percent tumor rejection in WT C57BL/6J mice transplanted with Y1.7AI and Y1.7LI melanoma cells and treated with mAlg8 or mLama4 NeoAg synthetic long peptide (SLP) plus poly I:C (pI:C) vaccines or pI:C alone starting on d. 3 post tumor-transplant and given every 6 days for total 3 doses. (**C**) Representative graphs displaying mAlg8 or mLama4 tetramer-specific CD8 T cells in Y1.7AI and Y1.7LI tumors treated with control mAb, anti-CTLA-4, pI:C, mAlg8 SLP + pI:C NeoAg vaccine (for Y1.7AI) or mLama4 SLP + pI:C NeoAg vaccine (for Y1.7LI) as in **A** and **B** and harvested on d. 16 post-tumor transplant. mAlg8-H2-K^b^, mLama4-H2-K^b^, or SIINFEKL-H2-K^b^ (irrelevant control) tetramers were labeled with PE and APC. Dot plots are gated on live CD45^+^ Thy1.2^+^ CD8 T cells. (**D**) Tumor growth, cumulative mouse survival, and percent tumor rejection in WT C57BL/6J mice transplanted with Y1.7LI melanoma cells and treated with control mAb, anti-CTLA-4, anti-PD-1, anti-CTLA-4 + anti-PD-1, irrelevant (for Y1.7LI) mAlg8 SLP + pI:C (Control VAX), or relevant mLama4 SLP + pI:C (neo VAX) starting on d. 7 post tumor-transplant, and subsequently on d. 10, 13, 16, 22, 28 for ICT and d. 13, 19 for NeoAg vaccines. Tumor growth data in (**A**), (**B**), and (**D**) are presented as individual mouse tumor growth as mean tumor diameter and are representative of (**A**) five, (**B**) three, or (**D**) four independent experiments. Tumor rejection graphs display cumulative percentage of mice with complete tumor rejection from independent experiments. Cumulative survival curves and tumor rejection graphs include mice from three independent experiments (**P < 0.01, ***P < 0.001, log-rank (Mantel–Cox) test). Bar graphs in (**C**), display mean ± SEM and are representative of at least three independent experiments (*P < 0.05, **P < 0.01, ***P < 0.005, NS, not significant; unpaired, two-tailed Student’s *t* test).

For this study, we modified the *Braf*^*V600E*^ *Pten^−/−^ Cdkn2a^−/−^* YUMM1.7 mouse melanoma line to express different combinations of MHC-I and MHC-II NeoAgs^38^. While GEMMs recapitulate many features of human cancers, GEMM-derived tumors generally nonimmunogenic and lack NeoAgs; however, they can be engineered to express NeoAgs to study tumor-immune interactions^19,39-43^. To generate a YUMM1.7 cell line expressing known tumor antigens, we engineered it to express minigenes encoding the G1254V mutation in Laminin subunit alpha 4 (mLama4^MHC-I^), the A506T mutation in Alpha-1,3-glucosyltransferase (mAlg8^MHC-I^), and the N710Y mutation in Integrin beta 1 (mItgb1^MHC-II^) NeoAgs in various combinations (Figure S1A). We generated YUMM1.7 lines expressing minigenes for mLama4^MHC-I^ + mItgb1^MHC-II^ (Y1.7LI) or mAlg8^MHC-I^ + mItgb1^MHC-II^ (Y1.7AI). Consistent with prior observations^38,44^, the parental YUMM1.7 melanoma line was insensitive to ICT, even anti-PD-1 and anti-CTLA-4 combination ICT (Figure S1B). In contrast, enforced expression of mLama4^MHC-I^ or mAlg8^MHC-I^ NeoAg along with mItgb1^MHCMHC-II^ NeoAg rendered YUMM1.7 melanoma lines (Y1.7LI and Y1.7AI) sensitive to anti-CTLA-4 ICT (Figure 1A).

We next asked whether therapeutic cancer vaccines composed of 10 μg of the SLP containing the minimal MHC-I NeoAg epitope and 50 μg of the adjuvant poly:IC (pI:C) could induce regression of the Y1.7LI and Y1.7AI NeoAg-expressing lines. Tumor bearing mice treated with pI:C alone displayed outgrowth of Y1.7LI or Y1.7AI melanoma, whereas vaccines composed of relevant NeoAg SLP + pI:C (neo VAX) induced complete rejection or delayed outgrowth of both Y1.7 NeoAg expressing variants (Figure 1B). Here the NeoAg vaccine-induced tumor rejection was dependent upon specific NeoAg expression, as vaccinating the Y1.7LI (mLama4-expressing) tumor bearing mice with the mAlg8 SLP + pI:C was not able to lead to tumor rejection and vice versa with the Y1.7AI (mAlg8-expressing) tumors (Figure 1B). Mice that rejected Y1.7AI or Y1.7LI tumors upon anti-CTLA-4 or neo VAX were rechallenged with the same tumors at least 60 days after rejection of primary tumors in the absence of any additional treatment. Upon secondary challenge, no detectable tumor was observed indicating long-term protection against rechallenge with the same tumor line (Figure S1C). In contrast, both Y1.7-NeoAg expressing lines grew out when injected in control naïve WT mice in the absence of treatment, indicating cell line preparations used in rechallenge experiments were capable of tumor formation. Additionally, when mice that previously rejected Y1.7LI tumors in response to anti-CTLA-4 or neo VAX therapy were rechallenged with the YUMM1.7 parental line, progressive tumor growth was observed (Figure S1D), indicating that long-term immunity was likely tumor NeoAg-specific.

We next used peptide-MHC (pMHC) tetramers to detect intratumoral CD8 T cells recognizing the mLama4 or mAlg8 NeoAg presented on H-2K^b^. Tumors from anti-CTLA-4 treated mice contained greater frequencies of mAlg8- or mLama4-specific CD8 T cells compared to the frequency of these cells in tumors from mice receiving control mAb (Figures 1C and S1E). Whereas pI:C alone had little effect on the NeoAg-specific CD8 T cell frequency, neo VAX induced an over 5-fold increase in the frequency of NeoAg-specific CD8 T cells (Figures 1C and S1E). This was particularly notable with the Y1.7LI tumor treated with NeoVAX, where over 14% of CD8 T cells were specific for mLama4. Neo VAX significantly increased the frequency of NeoAg-specific CD8 T cells co-expressing the inhibitory receptors PD-1 and TIM-3 (Figure S1F). Although PD-1 and TIM-3 are associated with dysfunction/exhaustion, their co-expression alone does not indicate reduced function and may be indicative of antigen stimulation and Tcell activation state^45,46^.

To expand on these observations, we focused on the Y1.7LI line, delayed treatment initiation until day 7, and evaluated anti-CTLA-4 and/or anti-PD-1. As expected, Y1.7LI tumor bearing mice treated with control mAb or control VAX (irrelevant mAlg8 SLP + pI:C) starting on day 7 post-transplant displayed progressive tumor outgrowth (Figure 1D). In contrast, anti-CTLA-4, anti-PD-1, combination anti-PD-1 plus anti-CTLA-4, or neo VAX induced tumor rejection in a majority of mice. ICT- and neo VAX-induced tumor rejection was dependent on both CD4 and CD8 T cells, as mAb depletion of either T cell subset completely abolished ICT efficacy (Figure S2A). Mice that rejected Y1.7LI tumors upon anti-PD-1 and/or anti-CTLA-4 or neo VAX initiated on day 7 were rechallenged with Y1.7LI at least 60 days after rejection of primary tumors in the absence of any additional treatment. Upon secondary challenge, no detectable tumor was observed under any of the conditions (Figure S2B). In contrast, Y1.7LI grew out when injected in control naïve WT mice in the absence of treatment.

### scRNAseq analysis of tumor microenvironment remodeling induced by NeoAg vaccines and ICT

**Figure 2.**
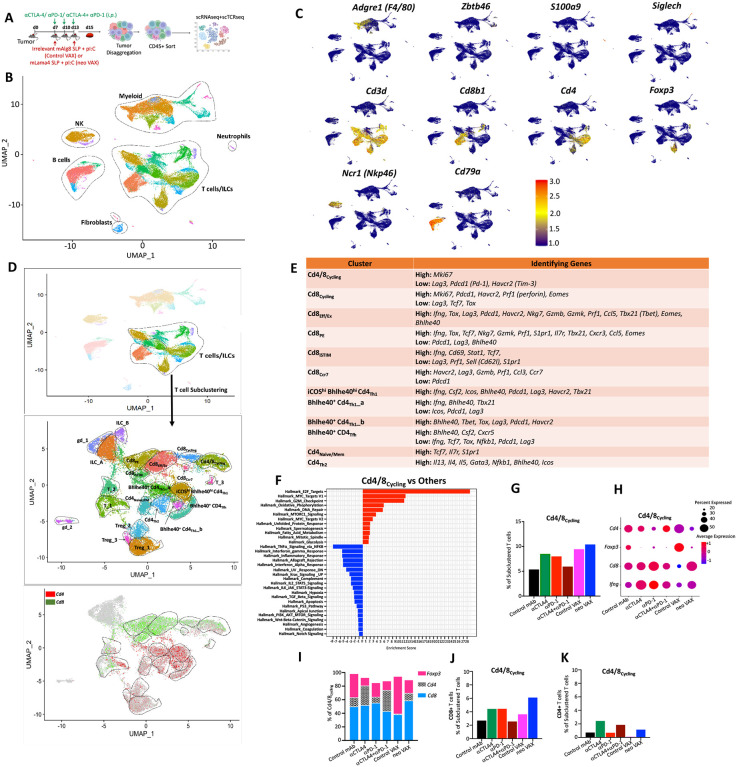
scRNAseq of Intratumoral Immune Cells from Y1.7LI Tumor Bearing Mice Treated with NeoAg Vaccines or ICT. (**A**) WT C57BL/6J mice were injected with Y1.7LI melanoma cells and subsequently treated beginning on d. 7 with control mAb, anti-CTLA-4, anti-PD-1, anti-CTLA-4 + anti-PD-1, irrelevant (for Y1.7LI) mAlg8 SLP + pI:C (Control VAX), or relevant mLama4 SLP + pI:C (neo VAX) and harvested on d. 15 post-tumor transplant. Intratumoral live CD45^+^ cells were sorted and analyzed by scRNAseq. (**B**) UMAP plot from scRNAseq of intratumoral CD45^+^ cells. Cell types were annotated based on lineage marker expression. (**C**) Feature plot showing lineage-specific transcripts defining lymphoid and myeloid cell types. (**D**) Feature plots displaying subclustering of activated T cell containing clusters and subclustered T cell/ILC cluster annotations (middle plot) and *Cd4* and *Cd8* expression (bottom plot). (**E**) Table showing select genes describing each CD8 and CD4 T cell cluster. (**F**) Gene set enrichment analysis (GSEA) displaying significantly enriched gene sets in cluster Cd4/8_Cycling_. (**G**) Proliferating T cells in cluster Cd4/8_Cycling_ by treatment condition represented as percentage of subclustered T cells. (**H**) Dot plot depicting expression level and percent of cells expressing *Foxp3, Cd4, Cd8, Ifng* in Cd4/8_Cycling_ by treatment condition. (**I**) Percentage of Foxp3^+^ CD4 Tregs, conventional CD4 T cells, or CD8 T cells in Cd4/8_Cycling_ by treatment condition. (**J**) Graph displaying CD8 T cells from cluster Cd4/8_Cycling_ represented as percentage of total subclustered T cells. (**K**) Graph displaying CD4 T cells from cluster Cd4/8_Cycling_ represented as percentage of total subclustered T cells.

We next used an unbiased approach to assess whether effective tumor-specific NeoAg vaccines induced TME alterations that are distinct or overlapping with different forms of ICT. Groups of Y1.7LI tumor bearing WT mice were treated with (1) control mAb, (2) anti-CTLA-4, (3) anti-PD-1, (4) anti-CTLA-4 + anti-PD-1, (5) control VAX (irrelevant SLP + pI:C), or (6) neo VAX (mLama4 SLP + pI:C) beginning on day 7 (Figure 2A). Tumors were harvested on day 15 (a critical timepoint prior to tumor rejection during ICT or neo VAX in this model) and live CD45^+^ cells were sorted and processed for 10X single cell 5' library generation and sequencing for scRNAseq. We performed unsupervised graph-based clustering and noted clusters of myeloid cells and lymphocytes (Figures 2B and 2C). scRNAseq and flow cytometry both indicated that immunotherapy altered the proportions of different myeloid and lymphoid subsets and in some cases these alterations were dependent upon the specific treatment employed (Figure S3A).

To gain more insights into how the different immunotherapies altered T cells in the TME, we chose clusters containing activated T cells for subclustering and identified multiple clusters of conventional CD4 and CD8 T cells, Foxp3^+^ CD4^+^ T regulatory cells (Tregs), gamma delta T cells (γδT), and innate lymphoid cells (ILCs) (Figures 2D, S3B-S3D, S4, and S5). Specific clusters of CD4 and CD8 T cells were annotated based on expression of select transcripts (Figures 2E, S4, and S5). We identified 5 exclusively CD8 T cell clusters, although this analysis did not distinguish their antigen specificity (Figures 2D, 2E, S4, S5, and S6A-S6F). Overall, these clusters of CD8 T cells spanned a range of activation states including proliferating (Cd8_Cycling_), CD69^hi^ IFN stimulated [Cd8_iSTIM_ (interferon STIMulated)], PD-1^+^ TCF7^+^ plastic/stem-like or progenitor exhausted (Cd8_PE_), and PD-1^+^ TCF7^−^ terminal effectors or dysfunctional/exhausted CD8 T cells (Cd8_Eff/Ex_) (Figures 2E, S4, S5, and S6A-S6F).

### NeoAg vaccines and ICT induce CD8 T cells with proliferative transcriptional signature

While most clusters contained either CD4 or CD8 T cells, cluster Cd4/8_cycling_ contained a mix of Tregs, CD4T cells, and CD8T cells and displayed a cell proliferation transcript signature (Figures 2D-2F S4 and S5). Not only did tumors from neo VAX, anti-PD-1, or anti-CTLA-4 treated mice have a greater frequency of cells within Cd4/8_Cycling_, but the ratio of cycling conventional CD4 and CD8 T cells to Tregs was higher as compared to control mAb or control VAX (Figures 2G-2K). Anti-CTLA-4 with or without anti-PD-1 reduced proliferating Tregs and expanded CD4 T cells within Cd4/8_Cycling_, while the ratio of proliferating CD8 T cells to Tregs or CD4 T cells was higher with anti-PD-1. Interestingly, neo VAX contained the greatest ratio of cycling CD8 T cells to other T cells in this cluster.

Cd8_Cycling_ also exhibited features of proliferation/cycling but was exclusively composed of CD8 T cells which displayed a more activated phenotype compared to Cd4/8_Cycling_, along with enrichment in gene sets associated with glycolysis, oxidative phosphorylation (OXPHOS), and fatty acid metabolism were enriched as manifested by Gene Set Enrichment Analysis (GSEA) (Figures S4, S5, S6A, and S6B). Whereas the percentage of Cd8_Cycling_ cells increased modestly with anti-CTLA-4 or anti-PD-1 ICT, neo VAX drove ~2-fold increase in the frequency of cells within this cluster (Figure S6B). These results suggest that while either neo VAX or ICT induce proliferating CD8 T cells, neo VAX more robustly expands subsets of proliferating CD8 T cells.

Cluster Cd8_Eff/Ex_ displayed little detectable *Tcf7* (encoding TCF-1) expression and elevated transcript expression of multiple inhibitory receptors and other genes associated with T cell activation, effector function, and also exhaustion/dysfunction including *Tox* (Figures S5, S6A, and S6C). Cd8_PE_ expressed *Pdcd1* (PD-1), but to less of an extent than Cd8_Eff/Ex_, and additionally expressed *Slamf6* and *Tcf7*, indicating a phenotype consistent with progenitor/precursor exhausted T cells that display plastic/stem-like properties (Figures S5, S6A, and S6D). neo VAX or monotherapy with anti-CTLA-4 or anti-PD-1 reduced the frequency of cells within Cd8_Eff/Ex_ and Cd8_PE_, whereas combination anti-CTLA-4 plus anti-PD-1 stood out as the only treatment to not decrease the frequency of Cd8_Eff/Ex_ (Figures S6C and S6D).

Within Cd8_Cycling_, Cd8_PE_, Cd8_iSTIM_, and Cd8_Ccr7_, the highest expression of *Lag3, Cd39*, and *Gzmb* within each respective cluster was observed with combination anti-CTLA-4 + anti-PD-1 ICT (Figures S5, S6A, S6B, and S6D-S6F). Additionally, *Prf1* was most robustly induced by combination ICT in all CD8 clusters, except for Cd8_Ccr7_, where neo VAX induced the highest expression (Figures S5 and S6A-S6F). Further, a pattern emerged within CD8 T cells whereby in each cluster, anti-CTLA-4 (alone or in combination with anti-PD-1), as well as neo VAX to some extent, drove higher expression of *Cd226* encoding the co-activating receptor CD226/DNAM-1. CD226 counteracting the actions of the inhibitory receptor TIGIT by competing for binding to ligands such as CD155^47^. Expression of *Tigit* followed an inverse pattern as *Cd226* with anti-CTLA-4 containing treatments and neo VAX reducing *Tigit* expression within clusters expressing the highest levels of *Tigit* (Cd8_Eff/Ex_, Cd8_Cycling_, Cd8_Ccr7_) (Figures S5, S6A, S6B, S6C, and S6F).

### Anti-PD-1 expands PD-1^+^ TCF7^−^ NeoAg-specific Teff/Tex, with combination anti-PD-1 + anti-CTLA-4 ICT inducing robust expansion of Bhlhe40^hi^ PD-1^+^ TCF7^−^ NeoAg-specific Teff/Tex

**Figure 3.**
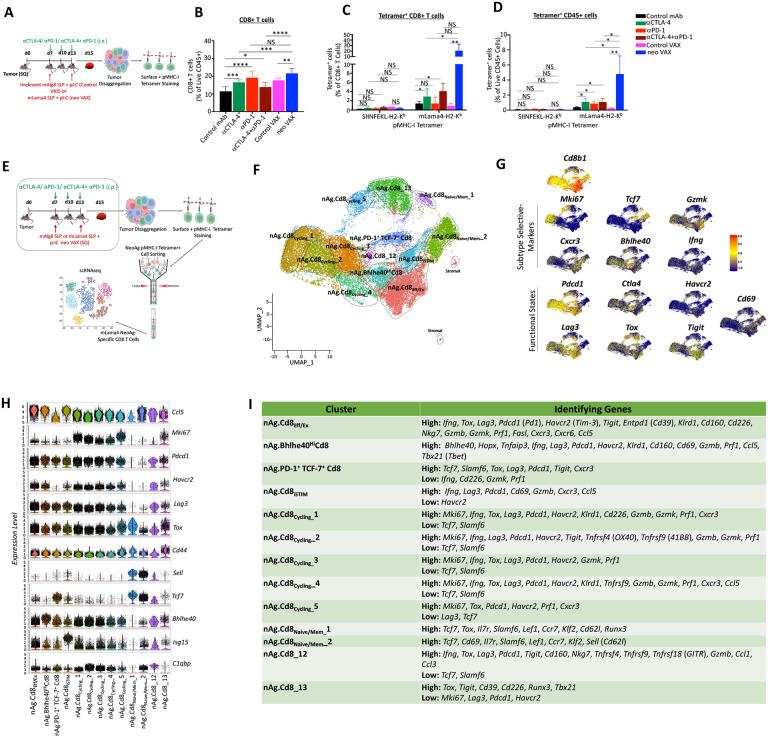
scRNAseq Profiling of mLama4 NeoAg-Specific CD8 T cells from Y1.7LI Tumor Bearing Mice Treated with NeoAg Vaccines or ICT. (**A**) WT C57BL/6J mice were injected with Y1.7LI melanoma cells and subsequently treated beginning on d. 7 with control mAb, anti-CTLA-4, anti-PD-1, anti-CTLA-4 + anti-PD-1, irrelevant (for Y1.7LI) mAlg8 SLP + pI:C (Control VAX), or relevant mLama4 SLP + pI:C (neo VAX) and harvested on d. 15 post-tumor transplant. single cell suspensions of harvested tumors from treated mice were stained with SIINFEKL or mLama4 PE and APC labelled tetramers and surface stained with flow antibodies. (**B**) Graph displaying CD8 T cells as a percentage of intratumoral live CD45^+^ cells in Y1.7LI tumors treated with control mAb, anti-CTLA-4, anti-PD-1, anti-CTLA-4 + anti-PD-1, irrelevant (for Y1.7LI) mAlg8 SLP + pI:C (Control VAX), or relevant mLama4 SLP + pI:C (neo VAX) and harvested on d. 15 post-tumor transplant. Graph displaying, (**C**) mLama4 tetramer-specific CD8 T cells and **(D)** mLama4 tetramer-specific CD45+ cells in Y1.7LI tumors treated with control mAb, anti-CTLA-4, anti-PD-1, anti-CTLA-4 + anti-PD-1, irrelevant (for Y1.7LI) mAlg8 SLP + pI:C (Control VAX), or relevant mLama4 SLP + pI:C (neo VAX) and harvested on d. 15 post-tumor transplant. mLama4-H2-K^b^ or SIINFEKL-H2-K^b^ (irrelevant control) tetramers were labeled with PE and APC. **(E)** WT C57BL/6J mice were injected with Y1.7LI melanoma cells and subsequently treated beginning on d. 7 with control mAb, anti-CTLA-4, anti-PD-1, anti-CTLA-4 + anti-PD-1, irrelevant (for Y1.7LI) mAlg8 SLP + pI:C (Control VAX), or relevant mLama4 SLP + pI:C (neo VAX) and harvested on d. 15 post-tumor transplant. Sorted mLama4 tetramer positive CD8 T cells were analyzed by scRNAseq. **(F)** UMAP plot from scRNAseq of mLama4 NeoAg-specific CD8 T cells. Cell types were annotated based on transcriptional activation states of NeoAg-specific CD8 T cells. **(G)** Feature plots displaying expression of select subtype or functional state defining genes. **(H)** Violin plots denoting expression level of select genes per neoAg-specific CD8 T cell. **(I)** Table showing select genes describing each NeoAg-specific CD8 T cells cluster.

We and others have demonstrated that tumor antigen-specific CD8 T cells have unique features as compared to bystander CD8 T cells and that immunotherapy primarily affects tumor antigen-specific versus bulk CD8 T cells^13,17,48-50^. Therefore, we monitored CD8 T cells specific for the mLama4 NeoAg in the setting of neo VAX or ICT (Figure 3A). neo VAX, anti-CTLA-4, anti-PD-1, or combination ICT all increased the overall frequency of intratumoral CD8 T cells (Figure 3B). Anti-CTLA-4 alone or in combination with anti-PD-1 drove a significant increase in the frequency of mLama4-specific CD8 T cells (Figures 3C, 3D, and S7A). Although anti-PD-1 did not alter mLama4-specific CD8 T cells as a percentage of CD8 T cells (Figures 3C and S7A), mLama4-specific CD8 T cells were significantly increased with anti-PD-1 when analyzed as a percentage of CD45^+^ cells (Figure 3D). Notably, neo VAX drove the greatest increase in mLama4-specific CD8 T cells from less than 2% of CD8 T cells in control mAb or control VAX to over 20% in mice treated with neo VAX, which corresponds to over 4% of all intratumoral CD45^+^ cells in mice treated with neo VAX (Figures 3C, 3D, and S7A). Since the scRNAseq profiling of intratumoral CD45^+^ cells did not distinguish NeoAg-specific CD8 T cells from other CD8 T cells, we profiled NeoAg-specific CD8 T cells by sorting intratumoral mLama4 tetramer positive CD8 T cells from mice treated with control mAb, anti-CTLA-4, anti-PD-1, anti-CTLA-4 plus anti-PD-1, control VAX, or neo VAX (Figure 3E). We profiled between 937 to 1762 mLama4-specific CD8 T cells for each of the different ICT treatment conditions and 4459, 6723, and 7646 mLama4-specific CD8T cells for control mAb, control VAX, and neo VAX, respectively. The two smallest clusters contained contaminating stromal cells, with the remaining clusters expressing transcripts consistent with CD8 T cells (Figure 3F, 3G, S7B, S7C, and S8). Apart from these two small clusters, this analysis uncovered multiple clusters of NeoAg-specific CD8 T cells that enabled us to distinguish features that were not evident when profiling bulk CD8 T cells.

**Figure 4.**
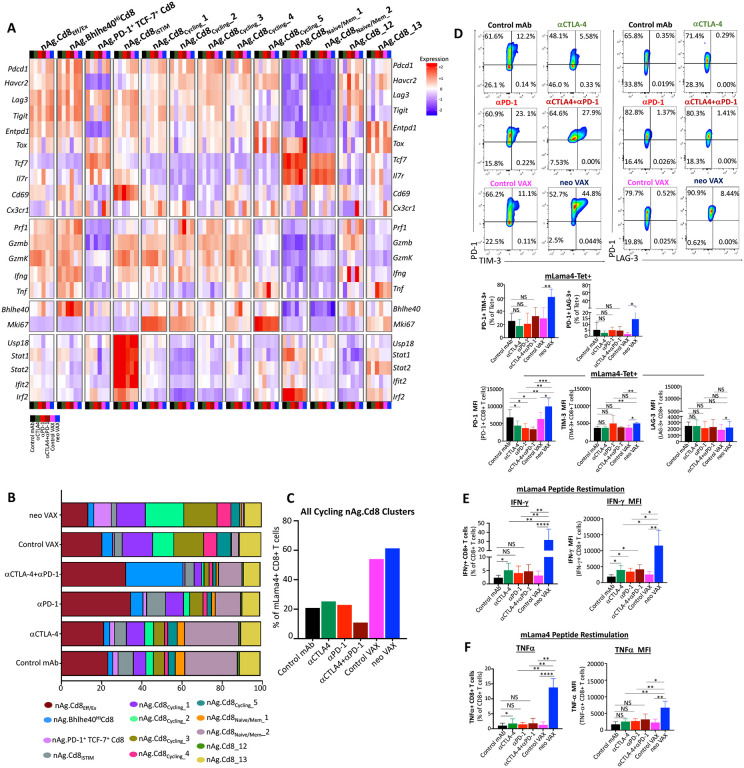
NeoAg Vaccines Promote Cycling and PD-1^+^ TCF-7^+^ Stem-Like NeoAg-Specific CD8 T Cells and Anti-CTLA-4 and/or Anti-PD-1 ICT Induce Distinct Effects on NeoAg-Specific CD8 T Cells. (**A**) Heat map displaying normalized expression of select genes in each NeoAg-specific CD8 T cell cluster by treatment condition. (**B**) Bar graphs displaying mLama4 NeoAg-specific CD8 T cells within each cluster by treatment condition. (**C**) The frequency of total mLama4 NeoAg-specific CD8 T cells within the combined 5 cycling clusters by treatment condition. **(D)** Representative flow cytometry plots displaying PD-1^+^ and/or TIM-3^+^/LAG-3^+^ after gating on mLama4 tetramer positive CD8 T cells and graphs displaying percent of PD-1^+^ TIM-3^+^/LAG-3^+^ or expression level of PD-1, TIM-3, or LAG-3 on PD-1^+^, TIM-3^+^, or LAG-3^+^ mLama4-specific CD8 T cells in Y1.7LI tumors treated with control mAb, anti-CTLA-4, anti-PD-1, anti-CTLA-4 + anti-PD-1, Control VAX, or neo VAX and harvested on d. 15 post-tumor transplant. Graph displaying **(E)** IFNγ^+^ mLama4-specific CD8 T cells and **(F)** TNFα^+^ mLama4-specific CD8 T cells assessed by intracellular cytokine staining of CD8 T cells isolated from Y1.7LI tumors under different treatment conditions and harvested on d. 15 post-tumor transplant. Bar graphs in **(D), (E)**, and **(F)** display mean ± SEM and are representative of at least three independent experiments (*P < 0.05, **P < 0.01, ***P < 0.005, **** *P* < 0.0001; NS, not significant, unpaired t test).

Clusters nAg.Cd8_Eff/Ex_ and nAg.Bhlhe40^Hi^Cd8 both highly expressed *Pdcd1, Havcr2, Lag3, Tigit*, and *Ccl5* as well as effector transcripts (*Nkg7, Gzmb, Gzmk, Prf1, Cxcr6*) and *Tox* and exhibited little to no detectable expression of *Tcf7* (Figures 3G-3I, 4A, S7B and S7C). neo VAX most notably reduced the proportion of nAg.Cd8_Eff/Ex_ cells, whereas the proportion of cells in this cluster increased with anti-PD-1 alone or in combination with anti-CTLA-4 (Figure 4B). The top defining marker of cluster nAg.Bhlhe40^Hi^Cd8 was *Bhlhe40* (Figures 3G, 3H, and S8), which we previously demonstrated was upregulated in tumor-specific T cells and required for CD4 and/or CD8 T cell effector function and response to ICT^20^. In addition to *Bhlhe40* (as well as *Pdcd1, Havcr2*, and *Lag3*) this cluster also expressed other transcripts induced by TCR activation, including *Ctla4, Cd69*, as well as *Nr4a1* (Nur77) and *Nr4a3* suggesting recent activation and/or TCR stimulation (Figures 3H, 4A, and S7B). nAg.Bhlhe40^Hi^Cd8 displayed the highest expression of *Tbx21* and *Ifng* amongst all the mLama4-specific CD8 T cells (Figure S7B). As compared to control mAb treatment, all other conditions (including control VAX) displayed a higher frequency of cells within this cluster, with the frequency of nAg.Bhlhe40^Hi^Cd8 increased from 2.4% of mLama4-specific CD8 T cells under control mAb conditions to 6.2% under anti-PD-1 treatment conditions (Figure 4B). Strikingly, anti-CTLA-4 and anti-PD-1 combination ICT increased this cluster to over 28% of mLama4-specific CD8 T cells.

### Anti-CTLA-4 alone or in combination with anti-PD-1 reduces expression of *Tox* and inhibitory receptors and promotes *Il7r* expression in PD-1^+^TCF7^−^ Teff/Tex NeoAg-specific CD8 T cells

In addition to increasing the frequency of cells with in PD-1^+^ TCF7^−^ Teff/Tex clusters (nAg.Cd8_Eff/Ex_ and nAg.Bhlhe40^Hi^Cd8), combination ICT increased expression of *Bhlhe40, Fasl, Il7r, Icos*, and *Cd28*, while decreasing *Tox, Pdcd1, Lag3, Entpd1*, and *Tigit* expression within both of these clusters (Figures 4A, S7B, and S7C). Further, combination ICT decreased expression on *Havcr2* and increased expression of *Cd69* within nAg.Bhlhe40^Hi^Cd8. While some of the features observed in combination ICT were distinct from either anti-CTLA-4 or anti-PD-1, within both these clusters the decrease in *Tox, Pdcd1, Lag3, Entpd1*, and *Tigit* (and *Havcr2* in nAg.Bhlhe40^Hi^Cd8) with combination ICT was also observed with anti-CTLA-4 ICT (but not with anti-PD-1) (Figures 4A, S7B, and S7C), suggesting that these specific changes included by combination therapy were primarily driven by anti-CTLA-4. In contrast, increased expression of *Bhlhe40* was most prominently observed in the presence of anti-PD-1. Other features (e.g., increased *Icos, Cd28*, and *Fasl* expression) were unique to the anti-CTLA-4 and anti-PD-1 combination ICT treatment conditions.

### NeoAg vaccination preferentially increases NeoAg-specific stem-like PD-1^+^TCF7^+^CD8 T cells and proliferating NeoAg-specific CD8 T cells

Amongst the most prominent NeoAg vaccine-driven features observed, NeoAg vaccines drove an over 3-fold increase in the frequency of mLama4-specific CD8 T cells within cluster nAg.PD-1^+^ TCF7^+^ Cd8 as compared to control mAb and over 8-fold increase as compared to control VAX (Figure 4B). Cluster nAg.PD-1^+^ TCF7^+^ Cd8 displayed high expression of *Slamf6* and *Pdcd1* amongst others; low to moderate expression of *Ifng, Gzmk, Prf1*, and *Cd226*; and no detectable expression of *Havcr2* (TIM-3) or *Entpd1* (CD39) (Figures 3G, 3H, 4A, and S7B). nAg.PD-1^+^ TCF7^+^ Cd8 also expressed transcripts encoding molecules related to T cell homing such as *Ccr7*, as well as *Bach2*^51^ and *Tcf7*. These features are consistent with CD8 T cells with plastic or stem-like properties or progenitor exhausted CD8 T cells. While NeoAg vaccines promoted this population, the proportion of NeoAg-specific CD8 T cells within this cluster was largely unchanged with anti-CTLA-4, reduced slightly with anti-PD-1, and even further reduced with combination anti-CTLA-4 and anti-PD-1 (Figure 4B). Anti-CTLA-4 containing treatments displayed decreased expression of *Pdcd1, Lag3, Tigit* and increased expression of transcripts encoding molecules related to T cell quiescence and homing such as *S1pr1, Sell* (Cd62l), and *Klf2*, as well as the IL-7 receptor transcript *Il7r* (Figures 4A, S7B, and S7C).

We annotated 5 clusters of NeoAg-specific CD8 T cells as “cycling”. NeoAg vaccination increased the frequency of cells in all 5 cycling NeoAg-specific CD8 T cell clusters displaying a range of activation states and proliferation signatures (Figures 4A, S7B, S7C, and S8). Each of the 5 cycling clusters also displayed a greater frequency of cells under control VAX conditions as compared to control mAb (Figure 4B). This suggests that although far more NeoAg-specific CD8 T cells are observed within tumors treated with neo VAX as compared to control VAX (Figures 3C and 3D), within NeoAg-specific CD8 T cells, pI:C contained in both control VAX and neo VAX likely promotes cycling of tumor-specific CD8T cells. These 5 cycling clusters together represented 20.9% of all mLama4-specific CD8 T cells under control mAb treatment, 54.1% under control VAX treatment, and 61.3% under neo VAX treatment (Figure 4C). Within nAg.Cd8_Cycling__1, nAg.Cd8_Cycling__2, and nAg.Cd8_Cycling__6, either control VAX or neo VAX increased the frequency of NeoAg-specific CD8 T cells to about the same level (Figure 4B). In contrast, nAg.Cd8_Cycling__3 represented 10.6% of NeoAg-specific CD8 T cells under control VAX conditions, whereas under neo VAX conditions, the frequency of cells within this cluster increased to 19.2% of NeoAg-specific CD8 T cells (Figure 4B). As compared to the other cycling clusters, nAg.Cd8_Cycling__3 expressed higher *Xcl1, Tnfrsf4* (OX40), *Tnfrsf9* (4-1BB), *Prf1*, and *Ifng* (Figures 4A, S7B and S8). The frequency of total cells within cycling clusters was modestly increased by anti-CTLA-4 or anti-PD-1 ICT, whereas anti-CTLA-4 plus anti-PD-1 combination ICT decreased the frequency by almost half.

### NeoAg vaccines induce robust expansion of NeoAg-specific IFN-γ^+^ CD8 T cells expressing PD-1 and LAG-3 and/or TIM-3

Since we noted that mice treated with neo VAX displayed a greater frequency of PD-1^+^ TIM-3^+^ NeoAg-specific CD8 T cells as compared to other treatments when treatment was initiated on d. 3 post-tumor transplant (Figure S1F), we assessed surface expression of PD-1, TIM-3, and additionally LAG-3 on intratumoral mLama4 NeoAg-specific CD8 T cells from mice when treatment initiation occurred on d. 7 post-transplant (as in our scRNAseq experiments). As expected, a majority of NeoAg-specific CD8 T cells expressed PD-1, with similar frequencies of PD-1^+^ TIM-3^+^ or PD-1^+^ LAG-3^+^ NeoAg-specific CD8 T cells observed between control mAb, control VAX, and the different ICT treatment conditions (Figure 4D). However, the expression level of PD-1, TIM-3, and LAG-3 on a per cell basis was lower in ICT treated groups. In contrast, a dramatic increase in the percentage of PD-1^+^ TIM-3^+^ or PD-1^+^ LAG-3^+^ mLama4-specific CD8 T cells was observed in mice treated with neo VAX and amongst PD-1^+^, TIM-3^+^, or LAG-3^+^ NeoAg-specific CD8 T cells, PD-1, TIM-3, and LAG-3, respectively, was expressed higher in the neo VAX treated group (Figure 4D). These results were consistent with the discordance between effective NeoAg vaccines and ICT in PD-1 and TIM-3 surface expression on NeoAg-specific CD8 T cells observed when treatment was initiated on day 3 (Figure S1F). Intracellular cytokine staining (ICS) on isolated intratumoral CD8 T cells restimulated with the mLama4 NeoAg peptide revealed anti-CTLA-4 increased the frequency of IFN-γ^+^ or TNFα^+^ CD8 T cells, while neo VAX induced the greatest expansion of more than 5-fold of IFN-γ^+^ or TNFα^+^ CD8 T cells (Figures 4E and 4F). Amongst IFN-γ^+^ CD8 T cells, expression of IFN-γ on a per cell basis increased significantly with anti-CTLA-4 and/or anti-PD-1, with the most robust increase occurring in mLama4 NeoAg-stimulated IFN-γ^+^ CD8 T cells isolated from neo VAX treated mice (Figure 4E).

### Anti-CTLA-4 promotes IFNγ^+^ Th1-like CD4 T cells expressing ICOS and Bhlhe40, while combination anti-CTLA-4 and anti-PD-1 ICT induces a small subset of Th2-like CD4 T cells

**Figure 5.**
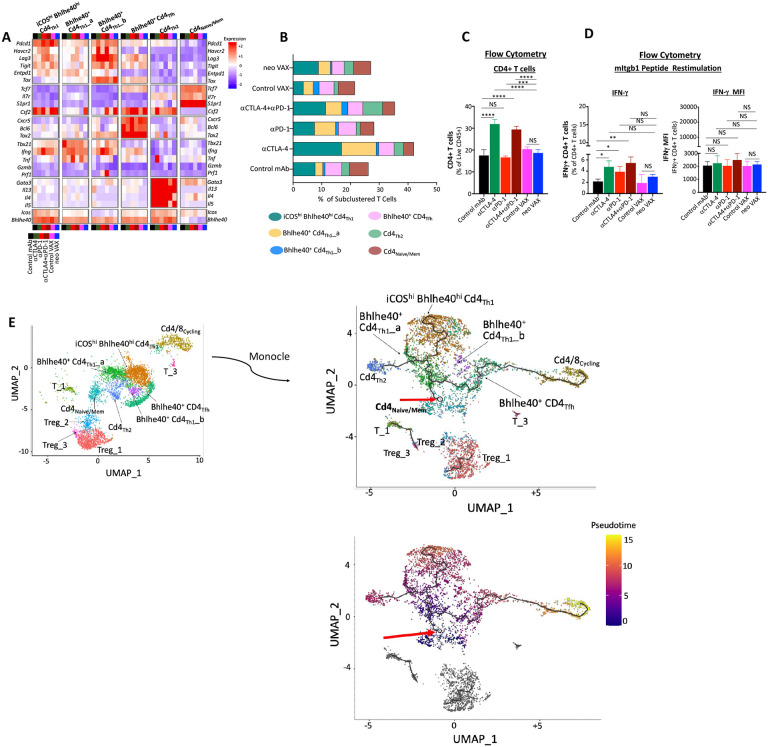
Anti-CTLA-4 Induces an ICOS^+^ Bhlhe40^+^ Th1-Like Subpopulation of CD4 T Cells and when Combined with Anti-PD-1, a Small Th2-Like Subpopulation. **(A)** Heat map displaying normalized expression of select genes in each CD4 T cell cluster. **(B)** Bar graphs depicting frequency of CD4 T cells within each cluster by treatment condition. **(C)** Graph displaying CD4 T cells as a percentage of intratumoral live CD45^+^ cells as determined by flow cytometry in Y1.7LI tumors under different treatment conditions and harvested on d. 15 post-tumor transplant. **(D)** Graph displaying IFNγ^+^ CD4 T cells as assessed by intracellular cytokine staining on CD4 T cells isolated from Y1.7LI tumors under different treatment conditions and harvested on d. 15 post-tumor transplant. **(E)** Monocle 3-Guided Cell Trajectory of CD4 T Cell Clusters. UMAP plot displaying exclusively CD4 T cell-containing clusters (left) of all experimental conditions, CD4 T cell trajectory graph overlaid on UMAP (middle) where the origin of the inferred pseudotime is indicated by the red arrow and assigned with pseudotime score 0, and geodesic distances and pseudotime score among other CD4 T cells are calculated from there based on transcripts associated cell states. CD4 T cell clusters overlaid on Monocle3 pseudotime plot (right). Bar graphs in **(C)** and **(D)** display mean ± SEM and are representative of at least three independent experiments (*P < 0.05, **P < 0.01, ***P < 0.005, ****P < 0.0001, NS, not significant, unpaired t test).

Since we observed that neo VAX or anti-CTLA-4/anti-PD-1 ICT required not only CD8 T cells, but also CD4 T cells for efficacy (Figure S2A), we examined CD4 T cells from our scRNAseq performed on sorted CD45^+^ cells (Figure 2A). Conventional CD4 T cells and Tregs were significantly altered by anti-CTLA-4, with anti-CTLA-4 inducing a higher frequency of CD4 T cells and reducing the percentage of Tregs as assessed by both scRNAseq and flow cytometry (Figures 2G-2I, 2K, S3A, and S3B). Anti-CTLA-4 (+/− anti-PD-1) induced notable increases in proliferating CD4 T cells and a decrease in proliferating Tregs within cluster Cd8/4_Cycling_ (Figures 2H, 2I, and 2K), further indicating that anti-CTLA-4 containing treatments dramatically affect the intratumoral CD4 T cell compartment. Most notably, anti-CTLA-4 (+/− anti-PD-1) induced subpopulations of Th1-like cells expressing *Ifng* and *Bhlhe40*, including cluster ICOS^hi^ Bhlhe40^hi^ CD4_Th1_ that also highly expressed *Icos* (Figures 2E, 5A, 5B, S5 and S9A). ICOS^hi^ Bhlhe40^hi^ CD4_Th1_ expressed transcripts indicative of highly activated T cells including high expression of *Pdcd1, Ctla4*, and the *Furin* transcript encoding a proprotein convertase, whose expression is regulated by TCR signaling and IL-12 signaling through STAT4^52^ (Figures 5A, S4, and S5). This cluster also expressed *Cxcr6, Csf2, Fasl*, and *Tnfaip3*, which encodes the A20 protein that regulates TCR/CD28-mediated NF-κB activation and TCR-mediated survival^53^ (Figure S5). ICOS^hi^ Bhlhe40^hi^ CD4_Th1_ displayed enrichment in IL-2 STAT5 and IL-6 JAK STAT3 signaling, TNFa signaling via NF-κB, and IFN-γ response gene sets amongst others (Figure S9A). neo VAX also exhibited a greater frequency of cells within this cluster as compared to control VAX (Figure 5B). Bhlhe40^+^ Cd4_Th1__a also expressed *Icos* and *Bhlhe40*, but to less of an extent than ICOS^hi^ Bhlhe40^hi^ CD4_Th1_ (Figures 5A and S5). Bhlhe40^+^ Cd4_Th1__a was further distinguished from ICOS^hi^ Bhlhe40^hi^ CD4_Th1_ by lower *Csf2, Runx3, Tnfaip3, Cxcr6, Furin, Pdcd1, Havcr2*, and *Lag3* expression and higher *Tbx21* (Tbet) expression. Anti-CTLA-4 dramatically increased the frequency of Bhlhe40^+^ CD4_Th1__a, with anti-PD-1 also increasing cells within this cluster (Figure 5B). Likewise, neo VAX increased the frequency of Bhlhe40^+^ Cd4_Th1__a cells but to much less of an extent (Figure 5B). Although both clusters expressed glycolytic enzyme transcripts, greater expression of several of these transcripts was seen in ICOS^hi^ Bhlhe40^hi^ CD4_Th1_, while Bhlhe40^+^ Cd4-_Th1__a displayed gene set enrichment in Fatty Acid Metabolism (Figures S5, S9A, and S9B). Additionally, both clusters displayed significant enrichment in TGF beta signaling gene sets (Figures S9A and S9B). Cluster Bhlhe40^+^ CD4_Th1__b was the smallest cluster of Th1-like cells and exhibited high *Ifng, Pdcd1, Tigit, Havcr2*, and *Lag3* expression (Figures 5A, S5, and S9C). This cluster also expressed the lowest level of *Icos* and the highest level of *Tox* amongst all CD4 clusters (Figures 5A and S5). While only subtle changes to the frequency of cells within this cluster were seen with treatments apart from control VAX and combination anti-CTLA-4 and anti-PD-1, with the latter displaying the highest frequency of cells within this cluster amongst all groups (Figure 5B).

Amongst all treatment conditions, mice treated with anti-CTLA-4 alone or in combination with anti-PD-1 displayed the highest expression of *Bhlhe40* and least expression of *Tnfrsf18 (GITR)* (Figures 5A, S5, S9A-S9E, and S9G). As observed within CD8 T cell clusters, within multiple CD4 clusters, anti-CTLA-4 alone or in combination drove an increase in *Cd226* expression and a decrease in *Tigit* expression, with neo VAX also following this same pattern but to less of an extent.

The increase in IFN-γ expressing Th1-like cells most prominently induced by anti-CTLA-4 was reflected by ICS on isolated intratumoral CD4 T cells restimulated *ex vivo* with the mItgb1 MHC-II NeoAg peptide. Anti-CTLA-4 +/− anti-PD-1 induced the most dramatic increase in both the overall frequency of conventional CD4 T cells, with anti-CTLA-4 and/or anti-PD-1 increasing the frequency of IFN-γ^+^ CD4 T cells upon restimulation with mItgb1 peptide (Figures 5C and 5D). This is in contrast to neo VAX, where only subtle changes were observed. Altogether, these findings indicate that while mice treated with anti-CTLA-4, alone or in combination with anti-PD-1, display the most dramatic increase in IFN-γ-producing Th1-like CD4 T cells within the tumor, anti-PD-1 also provokes IFN-γ^+^ CD4 T cells (Figure 5D). This is supported by comparing the expression of *Ifng* transcript within *Ifng*^+^ CD4 T cells clusters, where anti-PD-1 induced increased *Ifng* expression in these clusters, even those whose frequency was unaltered by anti-PD-1 (i.e., ICOS^hi^ Bhlhe40^hi^ Cd4_Th1_, Bhlhe40^+^ Cd4_Th1__b, Cd4_Tfh_) (Figures 5A, S5, and S9A-S9D).

Interestingly, combination ICT induced a small population of cells found in Cd4_Th2_, which also expressed *Icos* and *Bhlhe40*, but unlike the other ICOS^+^ Bhlhe40^+^ clusters the transcripts for *Ifng, Tim3*, and *Lag3* transcript were barely detectable (Figures 5A, S5, and S9E). Amongst CD4 clusters, Cd4_Th2_ expressed the highest level of *Cxcr6*, as well as *Tnfaip3* (Figure S5). In addition to low *Ifng* expression, many cells within Cd4_Th2_ expressed *Gata3, Il4, Il5*, and *Il13*, indicative of a Th2-like cluster (Figures 5A, S5, and S9F). Although cluster Cd4_Th2_ expressed Th2 cytokine transcripts and *Gata3*, both Cd4_Th2_ and ICOS^hi^ Bhlhe40^hi^ CD4_Th1_ highly expressed *Furin* and *Bhlhe40* (Figures 5A and S5). ICOS^hi^ Bhlhe40^hi^ CD4_Th1_ also displayed enrichment in TNFa signaling via NFkB and IFN-γ response gene sets, along with Kras signaling up, whereas Cd4_Th2_ displayed enrichment in Kras signaling down (Figures S9A and S9E).

To gain insight into the temporal dynamics of the observed changes in CD4T cells, we used Monocle to analyze scRNAseq data^54^. Monocle suggested that the starting point for conventional CD4 T cells corresponds to cells within either the Cd4_Naive/Mem_ cluster (expressing *Tcf7, Il7r*, and *S1pr1*, indicative of naïve or memory phenotype) or CD4 T cells within the Cd4/8_Cycling_ cluster (Figure 5E) with Cd4_Tfh_ connecting Cd4/8_Cycling_ CD4 T cells to the main trajectory towards Cd4_Naive/Mem_ and the branch to more activated, polarized CD4T cells. Notably, a pseudotime trajectory branch point occurs whereby activated CD4 T cells occupy Th1-like ICOS^hi^Bhlhe40^hi^Cd4_Th1_ driven by anti-CTLA-4 (+/− anti-PD-1) (and to a lesser extent by neo VAX) or encounter another branch whereby they assume one of two fates: they either become Th1-like CD4 T cells within Bhlhe40^+^Cd4_Th1__a or become Th2-like Cd4_Th2_, with Bhlhe40^+^ Cd4-_Th1__a being induced by anti-CTLA-4 and/or anti-PD-1 or neo VAX and Cd4_Th2_ primarily being driven by combination anti-CTLA-4 + anti-PD-1.

### Features of intratumoral Treg subpopulations during NeoAg vaccine or ICT treatment

We also identified three CD4 Foxp3^+^ Treg clusters (Figures S3B). Treg_1 and Treg_3 appeared to be the most activated with Treg_3 expressing the highest level of *Ctla4, Havcr2*, and *Klrg1* (Figure S5). Mice treated with anti-CTLA-4 alone or in combination with anti-PD-1 experienced a decrease in frequency of Treg_1 and Treg_3 (Figures S3B), which is consistent with previous results that the anti-CTLA-4 mAb we used (mouse IgG2b; clone 9D9) partially depletes Tregs, especially those highly expressing CTLA-4^55^. Treg_2 expressed lower amounts of *Ctla4, Havcr2, Tigit*, and *Klrg1* with the frequency of these Tregs not being affected by anti-CTLA-4, whereas anti-PD-1 with or without anti-CTLA-4, control VAX, or neo VAX displaying a greater frequency of cells in this cluster (Figure S3B). As compared to control VAX, the cellular density of Treg_1 and Treg_2 decreased in tumors from mice treated with neo VAX (Figures S3B). Further, transcript expression of *Foxp3* in Treg_2 was lower in the neo VAX group (Figure S3B). These alterations to the overall frequency of Tregs most prominently observed in the presence of anti-CTLA-4 are also corroborated by flow cytometry analysis (Figure S3A).

### Intratumoral myeloid cell compartment during NeoAg vaccines or ICT treatment

We first noted that neutrophils represented a small proportion of the overall immune infiltrate as more reliably shown by flow cytometry (Figures 2B and S3A). Consistent with previous observations in sarcoma models^18^, anti-CTLA-4 (+/− anti-PD-1) increased the proportion of neutrophils to just under 1.5% of CD45^+^ intratumoral cells (Figure S3A). To comprehensively characterize the signatures of intratumoral macrophages and DCs, we subclustered the myeloid component excluding the single neutrophil cluster (Figure S10A). In addition to a cluster of plasmacytoid DCs (pDCs), four other DC clusters were identified (CD103^+^ cDC1, CD63^+^ Ccr7^+^ cDC, Ccr7^+^ cDC, and Mgl2^+^ DC) (Figures S10A-S10G). Cluster CD103^+^ cDC1 expressed multiple classical DC (cDC) 1 transcripts including *Itgae* (*Cd103*), *Xcr1*, and *Clec9a* (Figures S10A and S10B). CD63^+^ Ccr7^+^ cDC and Ccr7^+^ cDC expressed *Ccr7, Cd1d1, Cd200, Fscn1, Cd274* (PD-L1), and *Pdcd1lg2* (PD-L2). These two migratory cDC clusters are likely composed of mregDCs, which describes a maturation state of cDC1s and cDC2s upon uptake of tumor antigen and although they express immunoregulatory molecules, they are not necessarily immunosuppressive^56,57^ (Figures S10B, S10D and S10E). CD63^+^ Ccr7^+^ cDC expressed higher *Cd63, Cd40, Cd70*, and *Btla* as compared to Ccr7^+^ cDC (Figures S10B and S10D).

### Distinct Macrophage Remodeling Induced by NeoAg Vaccines and ICT

**Figure 6.**
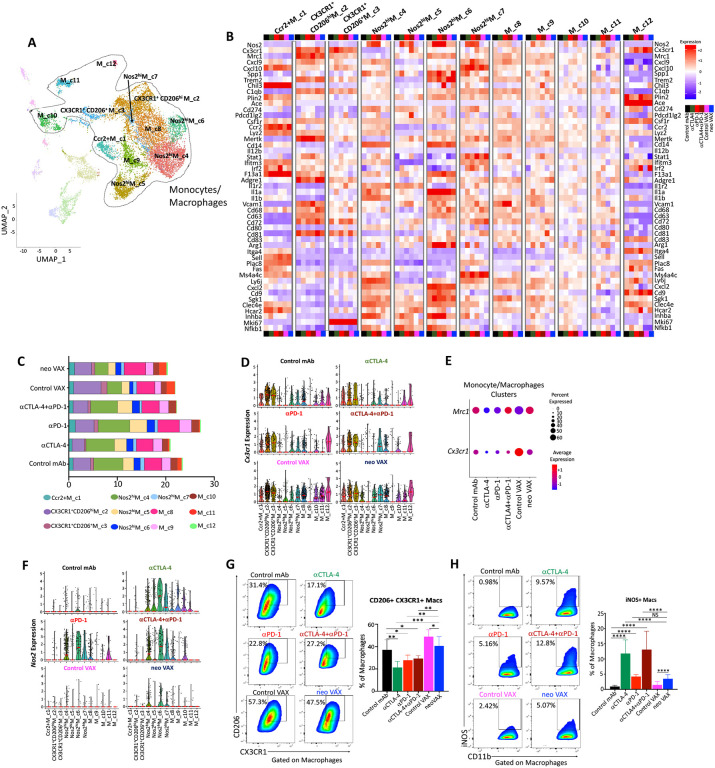
NeoAg Vaccines Promote Partially Distinct Macrophage Remodeling from ICT. **(A)** UMAP displaying sub-clustering of select myeloid clusters from CD45^+^ scRNAseq analysis (See Figure 2A). **(B)** Heatmap displaying normalized expression of select genes in each monocyte/macrophage cluster by treatment condition. **(C)** Percent monocytes/macrophages in each cluster by condition and treatment represented as percent of live CD45^+^ cells. **(D)** Violin plots denoting expression level of *Cx3cr1* transcript per cell in each monocyte/macrophage cluster by treatment condition. **(E)** scRNAseq dot plot depicting expression level/percent of cells expressing *Mrc1* and *Cx3cr1* within all monocytes/macrophages clusters by treatment condition. **(F)** Representative flow cytometry plots and graph displaying CX3CR1^+^CD206^+^ macrophages in Y1.7LI tumors under different treatment conditions and harvested on d. 15 post-tumor transplant. **(G)** Violin plots denoting expression level of *Nos2* (iNOS) transcript per cell in each monocyte/macrophage cluster by treatment condition. **(H)** Representative flow cytometry plots and graph displaying iNOS^+^ macrophages in Y1.7LI tumors under different treatment conditions and harvested on d. 15 post-tumor transplant. For flow cytometry analysis in **(F)** and **(H)**, dot plot displaying CX3CR1^+^CD206^+^ and iNOS^+^ macrophages are gated on macrophages using a gating strategy previously described (39). Bar graphs in **(F)** and **(H)** display mean ± SEM and are representative of at least three independent experiments (**P < 0.01, ****P < 0.0001, NS, not significant, unpaired *t* test).

We observed multiple intratumoral monocyte/macrophage clusters in Y1.7LI displaying a range of phenotypic states^58,59^ (Figures 6A, 6B, and S11). Ccr2^+^ M_c1 displayed transcripts consistent with monocytes, including *Ccr2* and *Chil3*, and the frequency of cells within this cluster only increased slightly with anti-PD-1 or neo VAX (Figures 6B, 6C, and S11). *Chil3*^*+*^ monocytes were previously shown to be reduced by a NeoAg vaccine in preclinical models^60^; however, the NeoAg vaccine and adjuvant used in that setting differed from ours.

We previously demonstrated that anti-CTLA-4 and/or anti-PD-1 induces macrophage TME remodeling characterized by a reduction in M2-like macrophages co-expressing the fractalkine receptor (CX3CR1) and the CD206 pattern recognition receptor and an increase in M1-like iNOS^+^ macrophages in mouse MCA sarcoma models^18,20^. We noted a similar ICT-induced remodeling trend in the Y1.7LI melanoma model. Whereas a slight decrease in the frequency of CX3CR1^+^ CD206^hi^ M_c2 cells expressing high levels of *Cx3cr1, Mrc1 (Cd206), Trem2, Vcam1, Cd63*, and *Cd72* was observed with anti-CTLA-4 and/or anti-PD-1 ICT, expression of *Cx3cr1* and frequency of *Cx3cr1*^+^ macrophages within this cluster was notably decreased under all ICT treatment conditions (Figures 6B-6D). The frequency of cells expressing *Cx3cr1* within this cluster under control VAX treatment was equal to or higher than with control mAb (Figure 6D). neo VAX reduced the proportion of cells within this cluster expressing *Cx3cr1* (Figure 6D). CX3CR1^+^ CD206^+^ M_c3 also expressed *Cx3cr1*, as well as *Mrc1 (Cd206), Trem2, Vcam1*, and *Cd72* with the latter transcripts being expressed less than in CX3CR1^+^ CD206^hi^ M_c2.CX3CR1^+^ CD206^+^M_c3 also displayed a cell proliferation transcript signature that included high expression of *Mki67* and exhibited lower *Mertk* expression as compared to CX3CR1^+^ CD206^hi^ M_c2 (Figure 6B). Anti-CTLA-4 alone reduced the frequency of CX3CR1^+^ CD206^+^ M_c3 (Figures 6C and 6D). Although the aforementioned two clusters expressed the highest levels of *Cx3cr1* and *Mrc1*, M_c8 and M_c9 macrophages also expressed *Cx3cr1* and *Mrc1* under control mAb conditions with ICT reducing expression of *Cx3cr1* within these clusters (Figures 6B and 6D). Comparable expression levels of *Cx3cr1* was observed in M_c8 under control VAX and neo VAX conditions, with neo VAX increasing the frequency of cells within this cluster (Figures 6B, 6C, and 6D). Under either control VAX or neo VAX conditions, M_c11 expressed both *Cx3cr1* and *Mrc1* and the frequency of cells within this cluster dramatically increased in mice treated with either control VAX or neo VAX, with ICT reducing this population (Figures 6B-6D). Overall, cells within all monocyte/macrophage clusters from mice treated with control VAX and neo VAX displayed higher expression of *Cx3cr1* as compared to ICT groups, with neo VAX also displaying similar expression of *Mrc1* as control mAb (Figure 6E).

Several monocyte/macrophage clusters expressed high levels of *Nos2* (iNOS); other clusters expressed varying levels of *Nos2*, with expression of *Nos2* being highly correlated with ICT treatment as well as neo VAX, but to less of an extent (Figures 6B and 6F). Further, expression of *Cd274* (PD-L1) also correlated with expression of *Nos2* within macrophage clusters, in particular under ICT treatment conditions (Figure 6B). While the overall frequency of these iNOS^+^ M1-like clusters often only modestly increased with ICT, the frequency of cells within these clusters expressing *Nos2* and/or *Nos2* expression on a per cell basis dramatically increased under all ICT conditions (Figures 6B, 6C, and 6F). Nos2^hi^M_c4 and Nos2^hi^ M_c6 both manifested high expression of *Nos2, Il1a, Il1b, Cxcl2, Inhba*, and *Nfkb1*, signatures of inflammatory macrophages^61^ (Figure 6B). While Nos2^hi^M_c4 displayed classic features of M1-like macrophages including low *Mrc1* expression, Nos2^hi^ M_c6 moderately expressed *Mrc1* as well as higher *F13a1, Trem2*, and *Il1a*^58,62^, along with lower *Il1r2* compared to Nos2^hi^M_c4 (Figure 6B). Nos2^hi^M_c4 displayed high expression of *Cxcl9* and *Spp1*, with expression of the latter diminished with ICT or neo VAX. Higher expression of *CXCL9* and lower *SPP1* expression was found to be correlated with macrophage prognostic score in cancer patients^63^. Nos2^hi^M_c5 highly expressed *Nos2* in the presence of ICT, with ICT also increasing the frequency of macrophages within this cluster (Figures 6B, 6C, and 6F). This cluster also expressed moderate levels of *Mki67* and other cell cycle related transcripts, indicative of iNOS^+^ macrophages with proliferative capabilities (Figure 6B). Nos2^hi^ M_c7 was the smallest iNOS^+^ macrophage cluster and in addition to *Nos2* expression, displayed highest expression of interferon-stimulated genes (ISGs) and also expressed high levels of *Cxcl9, Cxcl10, Cd274, Cd72, Cd81*, and *Ms4a4c* (Figures 6B and 6F). Although M_c8 and M_c9 expressed modest levels of *Cx3cr1* under control mAb conditions, its expression was reduced by ICT along with induction of *Nos2* expression within these clusters (Figures 6B, 6D, and 6F). These same overall patterns were manifested at the protein level where in anti-CTLA-4 and/or anti-PD-1 treated mice, the frequency of intratumoral CX3CR1^+^ CD206^+^ macrophages decreased with a concomitant increase in iNOS^+^ macrophages induced by ICT (Figures 6G and 6H). In contrast, while neo VAX treated mice also displayed a greater frequency of iNOS^+^ macrophages, CX3CR1^+^ CD206^+^ macrophages were only slightly reduced by neo VAX as compared to control VAX, but nonetheless were maintained at a similar frequency as seen in control mAb treated mice (Figures 6F and 6G). Since the frequency of CX3CR1^+^ CD206^+^ macrophages in both control VAX and neo VAX treated mice were similar or even greater than in control mAb treated mice, induction or maintenance of this macrophage subpopulation was likely linked to the pI:C adjuvant used in both control Vax and neo VAX. These results reveal that despite a relatively a similar abundance of M2-like CX3CR1^+^ CD206^+^ macrophages that were previously associated with progressively growing tumors in untreated or control mAb treated mice^18,20^, tumors in mice treated with neo VAX regress equivalently to ICT treated mice.

### ICT Broadens Therapeutic Window for Neoantigen Vaccines

**Figure 7.**
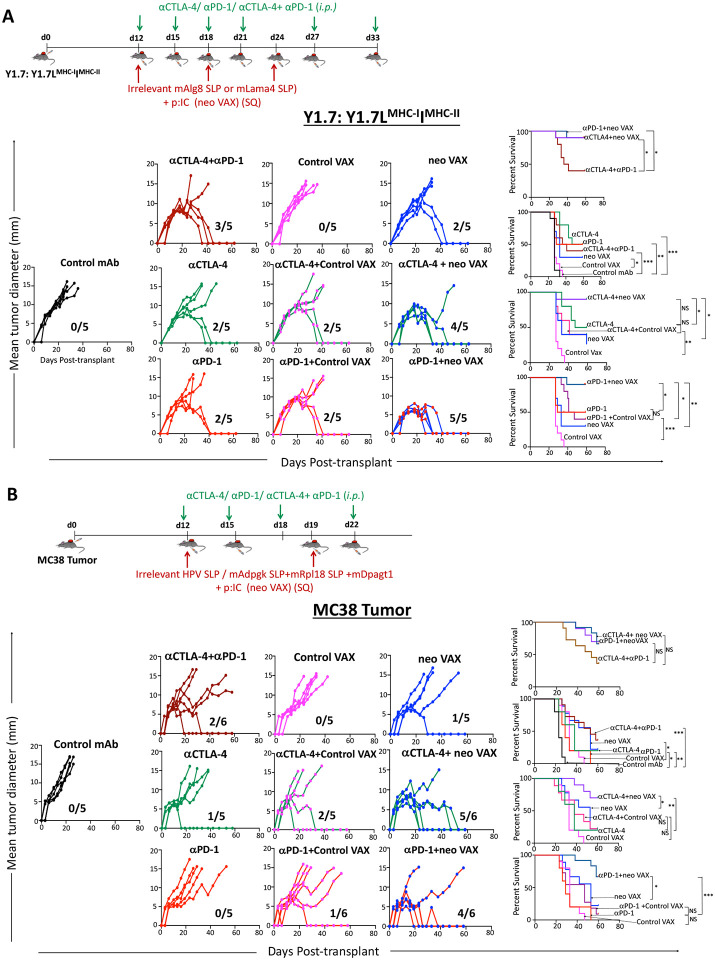
NeoAg Vaccines Broaden the Therapeutic Window for Anti-CTLA-4 or Anti-PD-1 ICT when Used in Combination Against Y1.7LI and MC38 Tumors. **(A)** Tumor growth and cumulative survival of WT C57BL/6J mice transplanted with Y1.7LI melanoma cells on d. 0 and treated beginning on d. 12 with different monotherapies: control mAb, anti-CTLA-4, anti-PD-1, irrelevant SLP + pI:C (Control VAX), or relevant mLama4 SLP + pI:C (neo VAX); or combination therapies: anti-CTLA-4 + anti-PD-1 combination ICT, anti-CTLA-4 + control VAX, anti-CTLA-4 + neo VAX, anti-PD-1 + control VAX, or anti-PD-1 + neo VAX. (B) Tumor growth and cumulative survival of WT C57BL/6J mice transplanted with MC38 cells on d. 0 and treated beginning on d. 12 with different monotherapies: control mAb, anti-CTLA-4, anti-PD-1, irrelevant HPV SLP + pI:C (Control VAX), or relevant mAdpgk SLP + mRpl18 SLP + mDpagt1 SLP + pI:C (neo VAX); or combination therapies: anti-CTLA-4 + anti-PD-1 combination ICT, anti-CTLA-4 + control VAX, anti-CTLA-4 + neo VAX, anti-PD-1 + control VAX, or anti-PD-1 + neo VAX. Tumor growth data in **(A)** and **(B)** are presented as individual mouse tumor growth as mean tumor diameter with fraction indicating # of mice rejecting tumor/# of mice used in experiment and are representative of three independent experiments. Cumulative survival curves in **(A)** and **(B)** include mice from three independent experiments (*P < 0.01, **P < 0.05, ***P < 0.001, log-rank (Mantel–Cox) test).

We noted changes that were not only shared between treatment conditions, but also distinct depending upon which treatment strategy was employed. Principle Component Analysis (PCA) further illustrated that neo VAX induces distinct changes to the immune TME as compared to anti-CTLA-4 and/or anti-PD-1 (Figure S12). This, together with our findings that neo VAX induces robust expansion of NeoAg-specific CD8 T cells that produce IFN-γ and appear functional, yet highly express PD-1/TIM-3/LAG-3 (Figures 3C, 3D, 4D, 4E, and S7A), prompted us to asked whether neo VAX could synergize with ICT. While neo VAX or ICT led to robust rejection of Y1.7LI when initiated on d. 7 post-transplant, a majority of tumor bearing mice displayed tumor outgrowth when treatment with anti-CTLA-4, anti-PD-1, or neo VAX was initiated on d. 12 post-transplant. We therefore used a d. 12 treatment start timepoint to assess whether combining neo VAX with anti-CTLA-4 or anti-PD-1 improved efficacy (Figure 7A). Mice treated with neo VAX in combination with anti-CTLA-4 or anti-PD-1 displayed enhanced tumor control as compared to control VAX (irrelevant SLP + pI:C) + anti-PD-1 or control VAX + anti-CTLA-4 (Figure 7A). Further, neo VAX used in combination with anti-CTLA-4 or anti-PD-1 provided superior tumor growth inhibition compared to combination anti-CTLA-4 and anti-PD-1 ICT. To extend our findings to a distinct tumor model, we assessed our vaccine protocol and combination treatment using the MC38 tumor model, which has several known endogenous MHC-I tumor NeoAgs^16,64,65^. Since it has been reported that expression of these NeoAgs varies depending on the source of the MC38 cell line, we previously confirmed in our MC38 line the presence of point mutations that form NeoAgs (mAdpgk, mRpl18, and mDpagt1)^16,64^. We assessed combinatorial treatments in MC38 tumor bearing mice by choosing an injection dose of cells (see Methods) and treatment schedule where monotherapy with anti-CTLA-4, anti-PD-1, or neo VAX alone is largely ineffective (Figure 7B). PBS, control VAX, or neo VAX was administered to MC38 tumor bearing mice on d. 12 and 19 post-transplant with or without anti-CTLA-4 or anti-PD-1 given on d. 12, 15, 18, and 22. Similar to results in the Y1.7LI model, neo VAX in combination with anti-CTLA-4 or anti-PD-1 provided superior protection versus monotherapy (Figure 7B). These findings in two distinct models complement ongoing NeoAg vaccine clinical trials and further support the rationale for combination NeoAg-based therapies.

## Discussion

In this study, we compared different immunotherapies that lead to tumor rejection (NeoAg SLP vaccines, anti-CTLA-4, anti-PD-1, anti-CTLA-4 + anti-PD-1, or NeoAg SLP cancer vaccines) and pertinent control treatments where tumor progression occurs using mouse melanoma models expressing defined NeoAgs and melanoma-relevant gain-of-function and loss-of-function genetic perturbations^38^. Although prior studies have examined NeoAg vaccines^60^, few (if any) studies have performed extensive comparisons between anti-PD-1, anti-CTLA-4, combination ICT, and NeoAg vaccines in the same robust experimental system. Further, while the focus of most prior studies involving ICT or NeoAg vaccines was on either lymphoid or myeloid cells^21,66^, our work has provided insights into both categories of cells and how different immunotherapies differentially affect these cells within the TME. Our treatment schedule and analyses were initially performed so that the NeoAg cancer vaccines or ICT we used lead to complete tumor rejection in a majority of mice; thus, we could compare and contrast the molecular and cellular changes that occur as a consequence of NeoAg vaccines or different forms of ICT and link them to outcomes. We specifically chose to study an SLP NeoAg vaccine as SLP vaccines are highly relevant with many clinical trials employing SLPs usually in combination with the adjuvant polyIC:LC vaccines are being conducted^7,9,67^.

The current study makes several key observations. First, NeoAg vaccines and ICT work by several overlapping mechanisms related to the CD8 T cell response, with key differences in the overall magnitude of the response and phenotype of NeoAg-specific CD8T cells observed. NeoAg vaccines induce the greatest expansion of functional intratumoral NeoAg-specific CD8T cells including proliferating T cells and PD-1^+^ TCF-1^+^ stem-like CD8 T cells and require not only CD8 T cells, but also CD4 T cells for efficacy. However, anti-CTLA-4 and/or anti-PD-1 also increased the frequency of intratumoral CD8 T cells, including NeoAg-specific CD8 T cells with enhanced production of IFN-γ. Anti-PD-1 alone, or most dramatically when administered in combination with anti-CTLA-4 ICT, induced a subset of Bhlhe40^hi^ NeoAg-specific CD8 T cells also display high expression of *Tbx21* and *Ifng*. Additionally, this subset expressed *Ctla4, Cd69*, as well as *Nr4a1* (Nur77) and *Nr4a3*, which suggest recent activation and/or TCR stimulation due to their known pattern of rapid and transient expression following T cell stimulation. Interestingly, a recent study identified Bhlhe40 as modulating a key differentiation point between progenitor and intermediate subsets of exhausted T cells in an in vitro exhaustion model and chronic LCMV infection^68^. While some of the features observed with combination ICT were distinct from either anti-CTLA-4 or anti-PD-1, the decrease in *Tox, Pdcd1, Lag3, Havcr2, Entpd1*, and *Tigit* with combination ICT was also observed with anti-CTLA-4 ICT, whereas the increased expression of Bhlhe40 within several NeoAg-specific CD8 T cell subsets was often more akin to the level of increase observed with anti-PD-1. These findings add to the accumulating evidence that the enhanced anti-tumor activity of combination anti-CTLA-4 and anti-PD-1 ICT is likely mediated by not only additive effects, but also through mechanisms distinct from the monotherapies^18,22^. In addition to modulating the CD8 T cell compartment, ICT notably impacted the CD4 T cell compartment as well. Anti-CTLA-4 reduced the frequency of Tregs and induced ICOS^+^ Th1-like conventional CD4 T cells displaying high expression of Bhlhe40, a transcription factor that we previously documented regulates T cell effector function during ICT^20^. Interestingly, subsets of Th1-like CD4 T cells with high expression of Bhlhe40 were previously found to be enriched in patients with microsatellite instability (MSI) colorectal cancer, who display favorable outcomes in response to anti-CTLA-4^69-71^. Further, studies in both preclinical models and human melanoma patients have revealed that anti-CTLA-4 induces ICOS^+^ CD4 T cells expressing IFN-γ^70,72^. Anti-PD-1 also increased the frequency of overall IFN-γ^+^ Th1-like CD4 T cells, but to less of an extent as compared to anti-CTLA-4. Combination anti-CTLA-4 and anti-PD-1 ICT induced a small, but significant subpopulation of Th2-like CD4 T cells (Cd4-_Th2_) expressing *Il4, Il13, Gata3*.

While vaccines targeting MHC-I NeoAgs predominately affected CD8 T cells, we found that these MHC-I NeoAg vaccines require CD4 T cells for efficacy. In MHC-II positive tumors, cytotoxic CD4 T cells have been show to directly kill tumor cells^73^; however, even with tumors lacking MHC-II expression, CD4 T cells are often required for anti-tumor immunity^19,42,74^. The importance of CD4 T cells may be due to their ability to produce cytokines such as IFN-γ and IL-2 and also likely stems from their ability to and their ability to be primed by cDC1s presenting tumor antigens and their CD40-dependent licensing of cDC1s that is critical for priming and activation of CD8 T cells^75,76^. Further, CD4T cell reprogramming of the myeloid compartment towards IFN-activated, iNOS-expressing tumoricidal and antigen-presenting phenotypes has also been implicated in tumor destruction^77,78^. The detailed mechanisms regarding the contribution of CD4 T cells in NeoAg vaccines targeting MHC-I NeoAgs remains to be fully elucidated by future studies and may entail multiple functions of CD4 T cells within both the lymph nodes and tumor. Although MHC-II NeoAgs are critical components of anti-tumor immunity, we specifically chose to utilize an SLP vaccine against a single MHC-I NeoAg in order to definitively link the MHC-I NeoAg vaccine to specific defined NeoAgs. Further, since MHC-II NeoAgs are more difficult to predict than MHC-I NeoAgs, we wanted to study the effects of an MHC-I NeoAg vaccine and whether this NeoAg vaccine approach in combination with anti-CTLA-4 or anti-PD-1 ICT could provoke rejection of larger, established tumors. SLPs offer several advantages over short peptides including the ability to specifically target professional APCs, stability, and the capacity to provoke both CD4 and CD8 T cells responses^79-81^; however, most immunogenic NeoAgs are either MHC-I or MHC-II NeoAgs, even when using SLPs. In our study, the SLP NeoAgs (mAlg8 or mLama4) provoked only NeoAg-specific CD8 T cell responses. Recent work revealed that physically linked MHC-I and MHC-II SLP vaccines provoked tumor growth inhibition in a preclinical squamous cell tumor model^82^. Determining whether incorporating an MHC-II NeoAg such as mItgb1 or even a shared, non-mutant antigen will enhance the efficacy of MHC-I NeoAg vaccines in our models is of future interest.

Beyond the T cell compartment, we noted a more divergent impact of NeoAg vaccines on the myeloid compartment than ICT. Both ICT and neo VAX increased M1-like iNOS^+^ macrophages, while ICT exclusively reduced the frequency of intratumoral CX3CR1^+^ CD206^+^ M2-like macrophages. Although less than in control VAX (irrelevant SLP + pI:C) treated mice, neo VAX (NeoAg SLP + pI:C) treated mice displayed a greater frequency of CX3CR1^+^ CD206^+^ macrophages as compared to control mAb or ICT treated mice. The detailed mechanisms by which control VAX and NeoAg vaccines induce CX3CR1^+^ CD206^+^ macrophages and the ability of NeoAg vaccines to provoke tumor regression in an environment that is partially distinct from that exhibited with ICT is yet to be fully delineated. In MCA sarcoma models, we previously found that intratumoral macrophage subpopulations displayed the spectrum of activation states ranging from an M2-like CX3CR1^+^ CD206^+^ phenotype in progressively growing tumors to a proinflammatory iNOS^+^ phenotype in tumors that will reject in response to ICT^18^, consistent with findings herein. Whereas induction of iNOS^+^ macrophages was dependent upon IFN-γ, ICT-driven depletion of CX3CR1^+^ CD206^+^ macrophages was partially independent of IFN-γ^18^. In our vaccine setting, we hypothesize that neo VAX comprising MHC-I NeoAg SLP and p:IC favors the induction of T cell-derived IFN-γ and other signals that drives monocyte polarization to iNOS^+^ macrophages upon entering the tumor, but other signals promote expansion or induction of CX3CR1^+^ CD206^+^ macrophages as well. These details of these signals are yet unknown but are likely induced by the pI:C (contained in both the control VAX and neo VAX), which acts as a TLR3 agonist in the endosome to potently induce a type IIFN response and can also activate RIG-I/MDA-5 in the cytosol to promote IL-12 production^83,84^. Although we and others use the term “M1-like” and “M2-like” to describe features that at least partially overlap with M1 or ‘classically’ activated and M2 or ‘alternatively’ activated macrophages, this is an oversimplification due to the complexity of activation and functional states of intratumoral macrophages^58^. Further, it is also important to note that although CX3CR1^+^ CD206^+^ macrophages display expression patterns consistent with immunosuppressive macrophages, CD206 alone is not sufficient to distinguish macrophages as immunosuppressive^59^, as we observed CD206 expression on some macrophages expressing iNOS. Nevertheless, it is tempting to speculate that combining NeoAg vaccines that maintain or promote CX3CR1^+^ CD206^+^ macrophages expressing high levels of *Trem2* with treatments targeting this macrophage population might enhance the efficacy of NeoAg vaccines.

Lastly, in both the Y1.7LI melanoma model and MC38 model, neo VAX combined with either anti-CTLA-4 or anti-PD-1 enhanced anti-tumor efficacy. Our rationale for assessing these combinations resulted from our observation that while some of the changes induced by neo VAX, as well as by ICT overlapped, distinct alterations were also noted. The unique features of each immunotherapy therefore prompted us to assess combining anti-CTLA-4 or anti-PD-1 with neo VAX and comparing efficacy to monotherapy or combination anti-CTLA-4 and anti-PD-1 ICT. We now find that combining anti-CTLA-4 or anti-PD-1 with neo VAX leads to better anti-tumor immune responses than even combination anti-CTLA-4 and anti-PD-1. While up to 20-30% of patients treated with anti-CTLA-4 or anti-PD-1 may experience durable cancer control, ~50% of metastatic melanoma patients treated with the combination of anti-CTLA-4 plus anti-PD-1 experience durable cancer control; however, immune related adverse events remain a problem^85,86^. As NeoAg vaccines have demonstrated favorable safety profiles thus far, combining NeoAg vaccines with single agent ICT may yield robust anti-tumor immunity with less toxicity than anti-CTLA-4 and anti-PD-1 combination ICT. While we find that anti-CTLA-4 or anti-PD-1 can synergize with neo VAX in different tumor models when we give the first NeoAg vaccine and ICT mAb at the same time, the timing of treatment may impact the response in different settings, as it has been shown that blockade of PD-1 in sub-primed CD8 T cells provokes PD-1^+^ CD38^+^ T cells that contribute to resistance to anti-PD-1 in other models and vaccine settings^87^. Although our approach of targeting a single NeoAg has revealed important insights, it is likely that targeting multiple NeoAgs and possibly even shared, non-mutant antigens will be required in patients due to tumor heterogeneity and therapy induced-immunoediting, with at least some of the antigens targeted by the vaccine needing to be clonal NeoAgs^88,89^.

This study provides key insights into the transcriptional, molecular, and functional changes that occur within major immune cell populations within the TME following different forms of cancer immunotherapy and compliments ongoing human clinical studies of NeoAg vaccines. Although we did not fully elaborate on every specific immune cell population we profiled, our analyses were designed to interrogate the entire immune TME, and thus our study should additionally provide an important resource. Therefore, the myeloid and lymphoid cell subsets and potential biomarkers we have described herein should inform the development of improved personalized NeoAg vaccines and combinatorial therapies in human patients.

## STAR Methods

Key resources Table S1

### Mice

All mice used were on a C57BL/6 background. WTC57BL/6J mice were purchased from Jackson Labs. All *in vivo* experiments used 8- to 12-week-old male or female mice (to match the sex and strain of the tumors). All mice were housed in a specific pathogen-free animal facility. All animal studies were performed in accordance with, and with the approval of the Institutional Animal Care and Use Committee (IACUC) of The University of Texas MD Anderson Cancer Center (Houston, TX).

### Plasmids

Gene blocks for mAlg8, mItgb1, or mLama4 were purchased from Integrated DNA Technologies. Minigene constructs were cloned into the BglII site of pMSCV-IRES GFP (mAlg8 and mItgb1) or pMSCV (mLama4 and mItgb1) using the Gibson Assembly method (New England Biolabs). To generate neoantigen-expressing Y1.7 melanoma cell lines, constructs were transiently transfected into Phoenix Eco cells using Fugene (Promega). After 48 hours, viral supernatants were filtered and subsequently used for transfection of Y1.7 melanoma cell line. Y1.7 mLama4 ^MHC-I^.mItgb1^MHC-II^ (Y1.7LI) and Y1.7 mAlg8 ^MHC-I^.mItgb1^MHC-II^ (Y1.7AI) were sorted based on GFP positivity and clones were verified for neoantigen expression.

### Tumor cell lines

The *Braf*^*V600E*^ *Cdkn2a*^−/−^ *Pten*^−/−^ YUMM1.7 parental line was originally generated in a male GEMM on the C57BL/6 background as described^38^. Parental YUMM1.7 was purchased from ATCC (CRL-3362) and was modified to generate NeoAg-expressing Y1.7 lines. The MC38 line was obtained from B. Schreiber (Washington University in St. Louis School of Medicine). All tumor cell lines were found to be free of common mouse pathogens and Mycoplasma as assessed by IDEXX IMPACT I mouse pathogen testing [PCR evaluation for: Corynebacterium bovis, Corynebacterium sp. (HAC2), Ectromelia, EDIM, Hantaan, K virus, LCMV, LDEV, MAV1, MAV2, mCMV, MHV, MNV, MPV, MTV, MVM, Mycoplasma pulmonis, Mycoplasma sp., Polyoma, PVM, REO3, Sendai, TMEV]. Tumor cell lines from the same cryopreserved stocks that were used in this study tested negative for Mycoplasma and were authenticated and found to be free of non-mouse cells as assessed by mouse cell STR profiling (IDEXX CellCheck mouse 19 plus Mycoplasma spp. testing).

### Tumor transplantation

The *Braf*^*V600E*^ *Cdkn2a*^−/−^ *Pten*^−/−^ YUMM1.7 parental melanoma line, Y1.7LI or Y1.7AI melanoma line, and the MC38 colorectal cancer line cells were propagated in R-10 plus BME media [RPMI media (HyClone) supplemented with 1% l-glutamine, 1% penicillin–streptomycin, 1% sodium pyruvate, 0.5% sodium bicarbonate, 0.1% 2-mercaptoethanol, and 10% heat-inactivated fetal calf serum (FCS) (HyClone) upon thawing, tumor lines were passaged 3 to 6 times before experimental use. Prior to injection, cells were washed extensively, resuspended at a concentration of 0.5 × 10^6^ cells (for YUMM1.7, Y1.7LI, and Y1.7AI) or 1.5 × 10^6^ cells (for MC38) in 150 μL of endotoxin-free PBS and 150 μL was injected subcutaneously into the flanks of recipient mice. Tumor cells were >90% viable at the time of injection as assessed by Trypan blue exclusion. Tumor growth was quantified by caliper measurements and expressed as the average of two perpendicular diameters. Lack of survival was defined as mouse death or mean tumor diameter size of 15 mm.

### Tumor rechallenge

For tumor rechallenge, mice that rejected primary tumors after treatment with anti-CTLA-4, anti-PD-1, anti-CTLA-4 + anti-PD-1, or NeoAg vaccines were then rechallenged with same number of cells used in primary challenge with either the same tumor line used in the primary tumor challenge or a different tumor line as indicated at least 60 days after complete rejection of the primary tumor.

### *In vivo* antibody treatments

For ICT treatment, YUMM1.7 parental, Y1.7LI, or Y1.7AI tumor-bearing mice were treated intraperitoneally with 200 μg of anti-CTLA-4 and/or anti-PD-1 on d. 3, 6, 9, 12, 18, and 22 or d. 7, 10, 13, 16, 22, and 28; or d. 12, 15, 18, 21, 27 and 33 post-tumor transplant. For controls, mice were injected with 200 μg of IgG2a isotype control antibodies. MC38 tumor-bearing mice were treated intraperitoneally with 200 μg of anti-CTLA-4 and/or anti-PD-1 on d. 12, 15, 18, and 22 post-transplant. For antibody depletion studies, 250 μg of control mAb, anti-CD4, or anti-CD8a was injected intraperitoneally into mice at d. −1 and every 7 days thereafter until day 20. CD4 and CD8 depletion was verified by flow cytometry analysis of surface-stained peripheral blood monocytes (PBMC) and intratumoral immune cells. For *in vivo* experiments, “*In vivo* Platinum”-grade antibodies that were verified to be free of mouse pathogens (IDEXX IMPACT I mouse pathogen testing) were purchased from Leinco Technologies: anti-PD-1 (rat IgG2a clone RMP1–14), anti-CTLA-4 (murine IgG2b clone 9D9), anti-CD4 (rat IgG2b clone GK1.5), anti-CD8a (rat IgG2b clone YTS169.4), and isotype controls (rat IgG2a clone 1–1, mouse lgG2a clone OKT3, or rat IgG2b clone 1–2).

### Peptides

Mutant Lama4 8-mer (VGFNFRTL), mutant Lama4SLP (QKISFFDGFEVGFNFRTLQPNGLLFYYT), mutant AdpgkSLP (HLELASMTNMELMSSIVHQ), mutant Rpl18SLP (KAGGKILTFDRLALESPK), mutant Dpagt1 SLP (EAGQSLVISASIIVFNLLELEGDYR), mutant Alg8 8-mer (ITYTWTRL), OVA-I_2577–264_ (SIINFEKL), mutant Itgb1 SLP (DDCWFYFTYSVNGYNEAIVHVVETPDCP), and OVA-II_323–339_ (ISQAVHAAHAEINEAGR) peptides were custom ordered from Peptide 2.0. All peptides were HPLC purified to >95% purity.

### Vaccination

Y1.7LI or Y1.7AI tumor bearing male mice were vaccinated subcutaneously with 10 μg mLama4 or mAlg8 synthetic long peptide (SLP) in combination with 50 μg of VacciGrade^™^ high molecular weight Polyinosinic-polycytidylic acid (pI:C) (InvivoGen) in a total volume of 150 μL diluted in endotoxin-free PBS on d. 3, 9, and 15 or d. 7, 13, and 19 or on d. 12, 18, and 24 post tumor transplant. MC38 tumor bearing female mice were vaccinated subcutaneously with 20 μg of mAdpgk SLP plus 20 μg of mRpl18 SLP plus 20 μg of mDpagt1 plus 50 μg pI:C adjuvant or control vaccine composed of 40 μg of irrelevant HPV SLP + 50 μg of pI:C on d. 12 and 19 post-tumor transplant. For SLP, peptide sequence used for mLama4; QKISFFDGFEVGFNFRTLQPNGLLFYYT (epitope underlined), for mAlg8; AVGITYTWTRLYASVLTGSLV (epitope underlined), for mAdpgk; HLELASMTNMELMSSIVHQ, for mRpl18; KAGGKILTFD***R***LALESPK and for mDpagt1; EAGQSLVISASIIVFNLLELEGDYR. mLama4 SLP served as a relevant SLP for the Y1.7LI line and an irrelevant SLP for the Y1.7AI line. mAlg8 served as a relevant SLP for the Y1.7AI line and an irrelevant SLP for the Y1.7LI tumor.

### Tetramers

OVA-I (SIINFEKL)-H-2K^b^(irrelevant control tetramer), mutant Alg8-H-2K^b^, and mutant Lama4-H-2K^b^ tetramers conjugated to PE or APC fluorophores, were obtained from the Baylor College of Medicine MHC Tetramer Production Facility.

### Tumor and spleen harvest

Established tumors were excised from mice, minced, and treated with 1 mg/mL type IA collagenase (Sigma-Aldrich) in HBSS (Hyclone) for 45 minutes at 37°C. Cells were washed thrice. Red blood cells were lysed using ACK lysis buffer (Gibco). To remove aggregates and clumps, cells were passed through a 40-μm strainer. Spleens were harvested, crushed, and vigorously resuspended to make single-cell suspensions. To remove aggregates and clumps, cells were passed through a 70-μm strainer and subsequently through a 40-μm strainer.

### TIL peptide restimulation

For peptide and PMA/ionomycin T-cell stimulation, cells from tumors, isolated as described above (see tumor and spleen harvest section), stained, and CD4 and CD8 T cells were sorted. For sorting CD4 and CD8 T cells, tumor cells were stained for 5 min at room temperature with 500 ng of Fc block (anti-CD16/32) and then stained with antibodies to CD45, CD3ε, CD4 or CD8α and Zombie NIR Viability dye in 100 μl of staining buffer. Cells were incubated for 30 minutes at 4°C. Live CD45^+^Cd3ε^+^CD4^+^ and live CD45^+^Cd3ε^+^CD8α^+^ were then sorted on a BD FACSAria II (BD Biosciences). Splenocytes harvested from naive mice and 100,000 splenocytes were then pulsed with 1 μM of various 8- or 9- or 17- or 28-mer peptides or simulated with 10 ng/mL of PMA (MilliporeSigma) and 1 μg/mL of ionomycin (Fisher) and 100,000 CD4 or CD8 TIL were subsequently added and incubated at 37 °C. Naive splenocytes added with or without CD4 or CD8 TIL, was included as control. After 1 h, BD GolgiPlug (BD Bioscience) was added in, and cells were incubated for an additional 5 h at 37 °C.

### Tetramer staining

For tetramer staining, cells were stained for 5 min at room temperature with 500 ng of Fc block (anti-CD16/32). H-2K^b^ tetramers conjugated to PE (1:50) or APC (1:100) for mutated Alg8, mutated Lama4, or SIINFEKL were added to cells and incubated for 20 min at 37°C. Tetramer-stained cells were further stained with surface antibody for anti-CD45, anti-Thy1.2, anti-CD8α, anti-CD4, anti-PD-1, anti-TIM-3, and anti-LAG-3 antibody for 20 min at 4 °C.

### Flow cytometry

For flow cytometry, cells were stained for 5 minutes at room temperature with rat anti-mouse CD16/32 (mouse BD Fc Block; clone 2.4G2, BD Biosciences) at 1 μg/million cells and then surface stained with flow antibodies for 20 minutes at 4°C. Surface antibodies were diluted in FACS staining buffer (PBS with 2% FCS, 2 mmol/L EDTA, and 0.05% NaN3; Sigma). Anti-mouse CD45-BV605, CD90.2/Thy1.2-PE-Cy7, anti-mouse CD8α-BV786, anti-mouse CD4-BV711, anti-mouse CD19-BV650, anti-mouse CD20-BV421, anti-mouse CD45R/B220-BBUV395, anti-mouse Nkp46/CD335-FITC, anti-mouse γδ TCR-PE-Cy7, anti-mouse PD-1-BV421, anti-mouse TIM-3, anti-mouse LAG-3-PerCP-Cy5.5, anti-mouse CD3ε-APC, anti-mouse CD64-BV421, anti-mouse Ly6G-Alexa Fluor 700, anti-mouse CX3CR1-FITC, anti-mouse I-A/I-E-BV650, anti-mouse CD103-BV421, anti-mouse CD24-BV711, anti-mouse CD11c-BV786, anti-mouse CD11b-APC, anti-mouse F4/80-BUV395, anti-mouse CD64-APC, CD117-FITC, anti-mouse CD11b- PerCP-Cy5.5 , anti-mouse PDCA-1/BST-2 BV650, anti-mouse CD172a APC, anti-mouse PDL1-PE, anti-mouse FcεRI-PE-Cy7 were used for surface staining at the indicated dilutions. Zombie NIR Viability dye was added at 1:500 during surface staining.

For intracellular staining, surface-stained cells were fixed and permeabilized with Fixation/Permeabilization Solution Kit (BD Bioscience). Fixed and permeabilized cells were then stained with anti-mouse Mrc1 (CD206)-PE-Cy7 and anti-mouse iNOS/NOS2-PE for 30 minutes at 4°C.

For FOXP3 staining, surface-stained cells were fixed and permeabilized using the eBioscience FOXP3/Transcription Factor Staining Buffer Set. Fixed and permeabilized cells were then stained with anti-mouse FOXP3-FITC for 30 minutes at 4°C.

For intracellular cytokine staining of lymphocytes, tumor cells were isolated and CD4 and CD8 T cells were sorted and added to peptide pulsed or PMA+Ionomycin stimulated splenocytes and incubated at 37°C for 6 hours with GolgiStop (BD Bioscience). Cells were then washed and stained for 5 minutes at room temperature with Fc block at 1 μg/million cells and then surface stained for 30 minutes at 4°C, and then fixed and permeabilized with BD Fixation and Permeabilization Kit. Fixed and permeabilized cells were then stained with anti-mouse IFNγ-APC, anti-mouse TNF-PE-Cy7 and anti-mouse Granzyme B-PE for 30 minutes at 4°C. All flow cytometry was performed on an BD Fortessa X-20, BD LSR, BD Fortessa, and analyzed using FlowJo software. Gating strategy used is depicted in Figure S13.

### scRNAseq

#### Antibody hashing

For analysis of NeoAg-specific CD8 T cells, cell and nuclei labeling were performed according to an adapted BioLegend cell hashing protocol (TotalSeq^™^-C Antibodies and Cell Hashing with 10x Single Cell 5' Reagent Kit v1.1 Protocol, BioLegend). Briefly, single cell suspensions of harvested tumors from treated mice were resuspended in BioLegend Cell Staining Buffer containing Fc receptor block and stained with mLama4 PE and APC labelled tetramers for 20 min at 37°C. Tetramer-stained cells from control mAb, control VAX, and neo VAX treatment conditions were immediately surface stained by adding anti-CD90.2/Thy1.2-PE-Cy7 and anti-CD8a-BV786 antibodies and incubating for 20 min at 4°C. Tetramer-stained samples from anti-CTLA-4, anti-PD-1, and anti-CTLA-4 plus anti-PD-1 treated groups were incubated with mixture of surface stain (anti-CD90.2/Thy1.2-PE-Cy7 and anti-CD8a-BV786 antibodies) and barcoded antibodies with unique hashtags for each treatment condition [anti-CTLA-4: Hashtag 1 Total Seq^™^-C0301 anti-mouse Hashtag 1 Antibody; anti-PD-1: Hashtag 2 (Total Seq^™^-C0302 anti-mouse Hashtag 2 Antibody); anti-CTLA-4 + anti-PD-1 combination: Hashtag 3 (Total Seq^™^-C0303 anti-mouse Hashtag 3 Antibody)]. Hashtag antibodies were used at a concentration of 1 μg per 2 million cells. Staining with surface antibodies and hashtag antibodies was done for 30 min at 4°C. Cells were then washed 3X with BioLegend Cell Staining Buffer. Sorted mLama4 tetramer-specific CD8 T cells with unique hashtags (anti-CTLA-4, anti-PD-1, and anti-CTLA-4 + anti-PD-1 samples) were pooled for single-cell library generation and CITE-seq (cellular indexing of transcriptomes and epitopes by sequencing) through multiplexing. Separate libraries were generated for control mAb, control VAX, and neo VAX samples and, thus, these were not multiplexed.

### scRNAseq library generation

Droplet-based 5 end massively parallel scRNAseq was performed by encapsulating sorted live CD45^+^ tumor-infiltrating cells into droplets and libraries were prepared using Chromium Next GEM Single-cell 5 Reagent Kit v2 (10x Genomics) according to manufacturer's protocol. The generated scRNAseq libraries were sequenced using an Illumina NovaSeq6000 S2 flow cell.

scRNAseq alignment, barcode assignment, and unique molecular identifier counting The Cell Ranger Single-Cell Software Suite available at https://support.10xgenomics.com/single-cell-gene-expression/software/overview/welcome was used to perform sample demultiplexing, barcode processing, and single-cell 5 counting. Cellranger mkfastq was used to demultiplex raw base call files from the NovaSeq6000 sequencer, into sample-specific fastq files. Files were demultiplexed with 81.9% to 97.1% perfect barcode match, and 90%+ q30 reads. Afterward, fastq files for each sample were processed with Cellranger count, which was used to align samples to mm10 genome, filtered, and quantified. For each sample, the recovered cells’ parameter was specified as 10,000 cells that we expected to recover for each individual library.

### Preprocessing analysis with Seurat package

The Seurat pipeline was applied to each dataset following tutorial specifications from https://satijalab.org/seurat/articles/archive; version 3.2 and https://hbctraining.github.io/scRNA-seq_online/. Data from all groups were merged into a single Seurat object, and integration was performed using the reciprocal principal component analysis (PCA) workflow to identify integration anchors. After integration, genes that were expressed in fewer than 3 cells and cells that contained fewer than 500 transcripts (unique molecular identifiers; UMI) were excluded. Cells with more than 10%) of mitochondrial transcripts were also excluded from analysis. The cutoffs used were set based on the characteristics of the cell population in each dataset. Data were normalized using LogNormalize method (counts for each cell divided by the total counts for that cell, multiplied by the scale factor of 10^4^ and natural-log transformed using log1p). PCA was performed on about 4,000 genes with PCA function. A uniform manifold approximation and projection (UMAP) dimensional reduction was performed on the scaled matrix (with most variable genes only) using the first 30 PCA components to obtain a two-dimensional representation of the cell states. For clustering, we used the function FindClusters that implements SNN (shared nearest neighbor) modularity optimization–based clustering algorithm on 30 PCA components, leading to 33 clusters.

Identification of cluster-specific genes and marker-based classification To identify marker genes, the FindAllMarkers function was used with likelihood-ratio test for single-cell gene expression. To characterize clusters, we used ImmGen database. For heatmap representation, mean expression of markers inside each cluster was used. To compare gene expression for the clusters inside cohorts (e.g., T cells, macrophages) we used FindMarkers function to calculate average log2 fold change and identify differentially expressed genes between each pair of experimental conditions using a Wilcoxon rank-sum test for calculating P values and Bonferroni correction for Padj values.

### T cell population analysis

To gain more insights into different immunotherapies-induced T cells remodeling in the TME, we subclustered activated T cells (excluding quiescent T cell clusters 10 and 12). Identification of most variable genes, PCA, UMAP, clustering, and marker selection analysis were performed as described above.

### Gene set enrichment analysis (GSEA)

To identify if MSigDB hallmark gene sets are up-regulated or down-regulated between clusters and treatments, we performed gene set enrichment analysis. Fold-changes of gene expression between comparisons were calculated using Seurat R package v.4.3.0.1, and normalized enrichment scores as well as p-values of given gene sets were then estimated using the gage R package v.2.46.1.

### Pseudo time trajectory analysis

To determine the potential lineage differentiation within CD4 T cell subpopulations, we used the Monocle3 R package to construct CD4 differentiation trajectories after specifying the corresponding cells as root nodes. Subsequently, graph test was used to find the pseudo time trajectory difference genes, and the obtained genes were used to plot the heat map.

### Statistical analysis

Samples were compared using an unpaired, two-tailed Student t test, two-way ANOVA, or log-rank (Mantel–Cox) test unless specified otherwise.

## Data and software availability

Data files for the sequencing data reported in this article will be deposited in the Gene Expression Omnibus (GEO) database and made publicly available at the time of publication. Software used in this study is available online: current version of Cell Ranger: https://support.10xgenomics.com/single-cell-gene-expression/software/downloads/latest; Seurat 4.0: https://satijalab.org/seurat/; ggplot2 3.3.3: https://ggplot2.tidy verse.org/index.html; and ImmGen: https://www.immgen.org. All other data generated in this study are available within the article and its Supplementary Data files, will be provided upon request at the time of publication, and/or will made publicly available at the time of publication via deposition in appropriate databases.

## Supplementary Material

Supplement 1 **Supplementary Figure 1. NeoAg Vaccines and ICT Induces Long-Term Tumor Protection in Y1.7AI and Y1.7LI Melanoma Models. (A)** Depiction of minigene NeoAgs used to express NeoAgs in the parental *Braf*^*V600E*^ *Pten*^−/−^ *Cdkn2a*^−/−^ YUMM1.7 melanoma line. mLama4 or mAlg8 and mItgb1 NeoAgs were separated by 2A peptides that induce ribosomal skipping during translation. **(B)** Tumor growth in WT C57BL/6J mice transplanted with parental *Braf*^*V600E*^ *Pten*^−/−^ *Cdkn2a*^−/−^ YUMM1.7 melanoma cells and treated with control mAb, anti-CTLA-4, anti-PD-1 or anti-CTLA4 + anti-PD-1 combination immune checkpoint therapy (ICT) on d. 3, 6, 9, 12, 18, 24 post tumor-transplant. **(C)** WT C57BL/6J mice were transplanted with Y1.7 mA^MHC-I^.mI^MHC-II^ (Y1.7AI) and Y1.7 mL^MHC-I^.mI^MHC-II^ (Y1.7LI) melanoma cells and treated with control mAb or anti-CTLA-4 on d. 3, 6, 9, 12, 18, 24 or mAlg8 NeoAg (relevant for Y1.7AI) synthetic long peptide (SLP) + poly I:C (pI:C) or mLama4 NeoAg (relevant for Y1.7LI) synthetic long peptide (SLP) + pI:C on d. 3, 9, 15. mice were rechallenged with same tumor used for initial tumor challenge at least 60 days post-rejection of primary tumor. Naïve WT C57BL/6J mice transplanted with Y1.7AI or Y1.7LI tumor without any treatment was included as control indicating cell line preps used in rechallenge experiments were capable of tumor formation. **(D)** WT C57BL/6J mice were transplanted with Y1.7LI melanoma cells and treated with anti-CTLA-4 ICT on d. 3, 6, 9, 12, 18, 24 or with mLama4 NeoAg SLP + pI:C on d. 3, 9, 15. Mice were rechallenged with either with same tumor used for initial tumor challenge (Y1.7LI) or parental *Braf*^*V600E*^ *Pten*^−/−^ *Cdkn2a*^−/−^ YUMM1.7 at least 60 days post-rejection of primary tumor. Naïve WT C57Bl6J mice transplanted with either Y1.7 LI or parental *Braf*^*V600E*^ *Pten*^−/−^ *Cdkn2a*^−/−^ YUMM1.7 without any treatment was included as control indicating cell line preps used in rechallenge experiments were capable of tumor formation. **(E)** Representative flow cytometry plots displaying mAlg8 or mLama4 tetramer-specific CD8 T cells in Y1.7AI and Y1.7LI tumors treated with control mAb, anti-CTLA-4, pI:C, mAlg8 SLP + pI:C NeoAg vaccine (for Y1.7AI) or mLama4 SLP + pI:C NeoAg vaccine (for Y1.7LI) and harvested on d. 16 post-tumor transplant. mAlg8-H2-K^b^, mLama4-H2-K^b^, or SIINFEKL-H2-K^b^ (irrelevant control) tetramers were labeled with PE and APC. Dot plots are gated on live CD45^+^ Thy1.2^+^ CD8 T cells. **(F)** Co-expression of PD-1 and TIM-3 on mAlg8- or mLama4-specific CD8 T cells in Y1.7AI and Y1.7LI tumors treated with control mAb, anti-CTLA-4, pI:C, mAlg8 SLP + pI:C NeoAg vaccine (for Y1.7AI), or mLama4 SLP + pI:C NeoAg vaccine (for Y1.7LI). Tumor growth data in **(B), (C)** and **(D)** are presented as individual mouse tumor growth as mean tumor diameter and are representative of three independent experiments.
**Supplementary Figure 2. NeoAg Vaccines and ICT Induce Long-Term Tumor Protection in Y1.7LI Melanoma Models in a T Cell-Dependent Manner. (A)** Y1.7LI tumor growth in WT C57BL/6J mice treated with control mAb, anti-CD4 or anti-CD8α mAbs on d. −1, 6, 13, 20 and anti-CTLA-4 or anti-PD-1 on d. 7, 10, 13, 16, 22, 28 or Irrelevant mAlg8 SLP + pI:C (Control VAX) or relevant mLama4 SLP + pI:C (neo VAX) on d. 7, 13, 19. **(B)** WT C57BL/6J mice transplanted with Y1.7LI melanoma cells and treated with control mAb, anti-CTLA-4, anti-PD-1, anti-CTLA-4 + anti-PD-1, irrelevant (for Y1.7LI) mAlg8 SLP + pI:C (Control VAX), or relevant mLama4 SLP + pI:C (neo VAX) starting on d. 7 post tumor-transplant, and subsequently on d. 10, 13, 16, 22, 28 for ICT and d. 13, 19 for NeoAg vaccines. Following post 60 days of primary tumor injection, mice were rechallenged with tumor used for initial tumor challenge. Naïve WT C57BL/6J mice transplanted with Y1.7 LI tumor without any treatment was included as control indicating cell line preps used in rechallenge experiments were capable of tumor formation. Tumor growth data in **(A)** and **(B)** are presented as individual mouse tumor growth as mean tumor diameter and are representative of three independent experiments.
**Supplementary Figure 3. scRNAseq Analysis of NeoAg Vaccines or ICT Induced Intratumoral Lymphoid and Myeloid Remodeling. (A)** Graph of flow cytometry data displaying intratumoral lymphoid and myeloid cells as a percentage of intratumoral live or live CD45^+^ cells in Y1.7LI tumors treated with control mAb, anti-CTLA-4, anti-PD-1, anti-CTLA-4 + anti-PD-1, irrelevant (for Y1.7LI) mAlg8 SLP + pI:C (Control VAX), or relevant mLama4 SLP + pI:C (neo VAX) beginning on d. 7 post-tumor transplant and harvested on d. 15. **(B)** Dot plot depicting expression level and percent of cells expressing *Foxp3, Ctla4, Icos, Tigit, Havcr2, Klrg1, Gzmb* and graph displaying frequency of regulatory T cell (Treg) clusters by treatment condition. **(C)** Graph displaying mixed T cell clusters represented as percentage of total subclustered T cells; GSEA displaying significantly enriched gene sets; and percentage of Foxp3^+^ CD4 Tregs, conventional CD4 T cells, or CD8 T cells in clusters T_1, T_2; and T_3 by treatment condition. **(D)** Graph displaying γδ T cell clusters represented as percentage of total subclustered T cells by treatment condition. **(E)** Graph displaying ILC clusters represented as percentage of total subclustered T cells by treatment condition. Bar graphs in **(A)** display mean ± SEM and are representative of at least three independent experiments (*P < 0.05, **P < 0.01, ***P < 0.005, ****P < 0.0001, NS, not significant, unpaired t test).
**Supplemental Figure 4. Heatmap displaying normalized expression per cell of top 10 cluster-defining genes for each T cell/ILC Cluster** (see Figure 2D).
Supplemental Figure 5. Heatmap displaying normalized expression of select genes in each T cell/ILC cluster by treatment condition.
Supplemental Figure 6. scRNAseq Analysis of Bulk CD8 T cells from Y1.7LI Tumor Bearing Mice Treated with NeoAg Vaccines or ICT. **(A)** Heat map displaying normalized expression of select genes in each bulk CD8 T cell clusters (see Figure 2A and 2D). **(B-F)** scRNAseq dot plot depicting expression level/percent of cells expressing select transcripts, GSEA displaying significantly enriched gene sets, and bar graphs depicting frequency of bulk CD8 T cells within each cluster by treatment condition.
Supplemental Figure 7. scRNAseq and Flow Cytometry Profiling of mLama4 NeoAg-Specific CD8 T cells from Y1.7LI Tumor Bearing Mice Treated with NeoAg Vaccines or ICT. **(A)** Representative flow cytometry plots displaying mLama4 tetramer-specific CD8 T cells in Y1.7LI tumors treated with control mAb, anti-CTLA-4, anti-PD-1, anti-CTLA-4 + anti-PD-1, irrelevant (for Y1.7LI) mAlg8 SLP + pI:C (Control VAX), or relevant mLama4 SLP + pI:C (neo VAX) and harvested on d. 15 post-tumor transplant. mLama4-H2-K^b^ or SIINFEKL-H2-K^b^ (irrelevant control) tetramers were labeled with PE and APC. Dot plots are gated on live CD45^+^ Thy1.2^+^ CD8 T cells. **(B)** Heatmap displaying normalized expression of select genes in each NeoAg-specific CD8 T cell clusters (see Figure 3E). **(C)** scRNAseq dot plot depicting expression level/percent of cells expressing select transcripts within each cluster by treatment condition.
Supplemental Figure 8. Heatmap displaying normalized expression per cell of top 10 cluster-defining genes for each NeoAg-specific CD8 T cells cluster (see Figure 3E).
Supplemental Figure 9. scRNAseq Profiling od CD4 T cells indicates Anti-CTLA-4 Induces an ICOS^+^ Bhlhe40^+^ Th1-Like Subpopulation of CD4 T Cells and when Combined with Anti-PD-1, a Small Th2-Like Subpopulation. **(A-E and G)** scRNAseq dot plots depicting expression level/percent of cells expressing select transcripts and GSEA displaying significantly enriched gene sets within each CD4 T cells cluster by treatment condition (see Figure 2D). **(F)** Violin plots denoting expression level of expression level of select genes per CD4 T cells.
Supplemental Figure 10. Dendritic Cell (DC) Changes Induced by ICT and NeoVAX in Y1.7LI Tumors. **(A)** UMAP displaying myeloid cell sub-clustering and DC annotations (See Fig. 2A and 6A). **(B)** Heatmap displaying normalized expression of select genes in each DC cluster. **(C-G)** scRNAseq dot plot depicting expression level/percent of cells expressing select transcripts and bar graphs depicting frequency of DCs within each cluster by treatment condition.
Supplemental Figure 11. Heatmap displaying normalized expression of select genes in each monocyte/macrophage cluster by treatment condition.
**Supplemental Figure 12. Principal Component Analysis of Subclustered T Cells/ILCs.** Each dot represents individual sample from different treatment conditions (see Figure 2A and 2D).
Supplemental Figure 13. Gating Strategy for Identifying Intratumoral Immune Cells. Flow cytometry dot plots and gating of intratumoral myeloid and lymphoid populations.

Supplement 2

## Highlights

- Neoantigen vaccines utilize distinct cellular mechanisms from anti-CTLA-4 or anti-PD-1 immune checkpoint therapy.
- Neoantigen vaccines preferentially induce PD-1+ TCF1+ stem-like and proliferating neoantigen-specific CD8 T cells
- Anti-PD-1 expands PD-1+ TCF-7- NeoAg-specific Teff/Tex, with combination anti-PD-1 + anti-CTLA-4 ICT inducing robust expansion of Bhlhe40hi PD-1+ TCF-7- NeoAg-specific Teff/Tex.
- Anti-CTLA-4 promotes Th1-like ICOS+ Bhlhe40+ CD4 T cells, while combination anti-CTLA-4 and anti-PD-1 ICT induces a small subset of Th2-like CD4 T cells
- Neoantigen vaccines induce partially distinct intratumoral macrophage remodeling from immune checkpoint therapy
- Neoantigen vaccines in combination with anti-CTLA-4 or anti-PD-1 provides enhanced tumor protection equivalent to or even exceeding protection seen with combination anti-CTLA-4 and anti-PD-1
