# Supplementary material for: Neoantigen Cancer Vaccines and Different Immune Checkpoint Therapies Each Utilize Both Converging and Distinct Mechanisms that in Combination Enable Synergistic Therapeutic Efficacy
Source: bioRxiv. 2024 Jan 26:2023.12.20.570816. Originally published 2023 Dec 22. Preprint. [Version 2] doi: 10.1101/2023.12.20.570816 (PMC10769249; doi:10.1101/2023.12.20.570816)
Supplement: Supplement 2 [file media-2.pdf]

REAGENT or RESOURCE	SOURCE	IDENTIFIER
***In vivo* Antibodies**		
Anti-CTLA-4 (clone 9D9)	Leinco	Cat# C2856, RRID: AB_2829611
Anti-CD279 (PD-1) (clone RMP1-14)	Leinco	Cat# RMP1-14, RRID: AB_2749820
Anti-mouse CD8 (clone YTS 169)	Leinco	Cat# C2442, RRID: AB_2829540
Anti-mouse CD4 (Clone GK1.5)	Leinco	Cat# C2838, RRID: AB_2829596
Mouse IgG2a Isotype	Leinco	Cat# P381, RRID: AB_2831654
**Flow Antibodies and reagents**		
Anti-CD16/32 (clone 2.4G2)	BD Biosciences	Cat#553141, RRID: AB_394656
Anti-mouse CD45 BV605 (clone 30-F11) (1:800 dilution)	BioLegend	Cat#103140, RRID: AB_2562342
Anti-mouse CD90.2/Thy1.2-PE-Cy7 (clone 30-H12) (1:200 dilution)	BioLegend	Cat#105326, RRID: AB_2201290
Anti-mouse CD8a-BV786 (clone 53–6.7) (1:200 dilution)	BD Bioscience	Cat#563332, RRID: AB_2721167
Anti-mouse CD4-BV711(clone RM4–5) (1:200 dilution)	BioLegend	Cat#100550, RRID: AB_2562099
Anti-mouse CD19-BV650 (clone 1D3) (1:200 dilution)	BD Bioscience	Cat#563235, RRID: AB_2738085
Anti-mouse CD20-BV421 (clone SA275A11) (1:200 dilution)	BioLegend	Cat#150405, RRID: AB_2566540
Anti-mouse CD45R/B220-BUV395 (clone RA3-6B2) (1:200 dilution)	BD Bioscience	Cat# 563793, RRID: AB_2738427
Anti-mouse Nkp46/CD335-FITC (clone 29A1.4) (1:300 dilution)	BioLegend	Cat# 560756, RRID: AB_1727465
Anti-mouse γδ TCR-PE-Cy7 (clone GL3) (1:300 dilution)	BioLegend	Cat# 118124, RRID: AB_11204423
Anti-mouse PD-1-BV421 (clone 29F.1A12) (1:200 dilution)	BioLegend	Cat# 135218, RRID: AB_2561447
Anti-mouse TIM-3 (clone RMT3-23) (1:200 dilution)	BioLegend	Cat# 119727, RRID: AB_2716208
Anti-mouse LAG-3-PerCP-Cy5.5 (clone C9B7W) (1:200 dilution)	BioLegend	Cat# 125212, RRID: AB_2561517
Anti-mouse CD3e-APC (clone 145–2C11) (1:200 dilution)	BioLegend	Cat# 100312, RRID: AB_312677
Anti-mouse CD64-BV421 (clone X54–5/7.1) (1:200 dilution)	BioLegend	Cat# 139309, RRID: AB_2562694
Anti-mouse Ly6G-Alexa Fluor 700 (clone 1A8) (1:400 dilution)	BD Biosciences	Cat# 127622, RRID: AB_10643269
Anti-mouse CX3CR1-FITC (clone SA011F11) (1:1,000 dilution)	BioLegend	Cat# 149020, RRID: AB_2565703

Anti-mouse I-A/I-E-BV650 (clone M5/114.15.2) (1:3,000 dilution)	BD Bioscience	Cat# 563415, RRID: AB_2738192
Anti-mouse CD103-BV421 (clone 2E7) (1:200 dilution)	BioLegend	Cat# 121422, RRID: AB_2562901
Anti-mouse CD24-BV711 (clone M1/69) (1:500 dilution)	BD Bioscience	Cat# 563450, RRID: AB_2738213
Anti-mouse CD11c-BV786 (clone HL3) (1:400 dilution)	BD Biosciences	Cat# 563735, RRID: AB_2738394
Anti-mouse CD11b-APC (clone M1/70) (1:400 dilution)	BioLegend	Cat# 101212, RRID: AB_312795
Anti-mouse F4/80-BUV395 (clone T45–2342) (1:400 dilution)	BD Biosciences	Cat# 565614, RRID: AB_2739304
Anti-mouse CD64-APC (clone X54–5/7.1) (1:400 dilution)	BioLegend	Cat# 139306, RRID: AB_11219391
Anti-mouse CD117-FITC (clone ACK2) (1:100 dilution)	BioLegend	Cat# 135115, RRID: AB_2561633
Anti-mouse CD11b- PerCP-Cy5.5 (clone M1/70) (1:200 dilution)	BioLegend	Cat# 561114, RRID: AB_394002
Anti-mouse PDCA- 1/BST-2 BV650 (clone 927) (1:200 dilution)	BD Biosciences	Cat# 747605, RRID: AB_2744173
Anti-mouse CD172a APC (clone P84) (1:200 dilution)	BioLegend	Cat# 144014, RRID: AB_2564061
Anti-mouse CD274/PDL1-PE (clone MIH5) (1:200 dilution)	BD Biosciences	Cat# 558091, RRID: AB_397018
Anti-mouse FcεRI-PE-Cy7 (clone MAR-1) (1:200 dilution)	BioLegend	Cat# 134326, RRID: AB_2572064
Anti-mouse Mrc1 (CD206)-PE-Cy7 (clone C068C2) (1:400 dilution)	BioLegend	Cat# 141720, RRID: AB_2562248
Anti-mouse FOXP3-FITC (clone FJK-16s)	eBioscience™	Cat# 11-5773-82, RRID: AB_465243
Anti-mouse IFNγ-APC (Clone XMG1.2) (1:200 dilution)	BD Biosciences	Cat# 554413, RRID: AB_398551
Anti-mouse TNF -PE-Cy7 (Clone MP6-XT22) (1:200 dilution)	BD Biosciences	Cat# 561062, RRID: AB_398553
Anti-mouse Granzyme B-PE (Clone NGZB) (1:200 dilution)	eBioscience™	Cat# 12-8898-82, RRID: AB_10870787
Anti-mouse iNOS/Nos2 PE (clone CXNFT) (1:200 dilution)	eBioscience™	Cat# 12-5920-82, RRID: AB_2572642
Zombie Fixable Viability™ Sampler Kit	BioLegend	Cat# 423105
**Chemicals, Peptides, and Gene blocks**		
RPMI-1640	HyClone	Cat# SH30096.02
Trypsin	Genclone	Cat# 25200056

Defined fetal bovine serum	HyClone	Cat# SH30070.03HI
HBSS	Hyclone	Cat# SH30588.02
PBS	Gibco	Cat# 20012027
Sodium Bicarbonate	Gibco	Cat# 25080094
Sodium Pyruvate	Gibco	Cat# 11360070
L-Glutamine	Gibco	Cat# A2916801
ACK lysis buffer	Gibco	Cat# A1049201
Phorbol-12-myristate-13-acetate (PMA)	MilliporeSigma	Cat# 500582
Ionomycin	Fisher	Cat# BP2527-1
Fugene	Promega	Cat# E2311
pMSCV-IRES GFP	addgene	Cat# 20672
Collagenase Type IA	Sigma-Aldrich	Cat# C9891
Poly(I:C) HMW VacciGrade™	InvivoGen	Cat# vac-pic
Gibson Assembly® Cloning Kit	NEB	Cat# E5510S
Mutant Lama4 peptide, sequence VGFNFRTL	Peptide 2.0	Custom order
Mutant Alg8 peptide, sequence ITYTWTRL	Peptide 2.0	Custom order
Mutant Lama4 SLP, sequence QKISFFDGFEVGFNFRTLQPNGLLFYYT	Peptide 2.0	Custom order
Mutant Adpgk SLP, sequence HLELASMTNMELMSSIVHQ	Peptide 2.0	Custom order
Mutant Rpl18 SLP, sequence KAGGKILTFD***R***LALESPK	Peptide 2.0	Custom order
Mutant Dpagt1 SLP, sequence EAGQSLVISASIIVFNL***L***ELEGDYR	Peptide 2.0	Custom order
OVA-I$_{257–264}$ peptide, sequence SIINFEKL	Peptide 2.0	Custom order
Mutant Itgb1 SLP, sequence DDCWFYFTYSVNGY***N***EAIVHVVETPDCP	Peptide 2.0	Custom order
OVA-II$_{323–339}$, sequence ISQAVHAAHAEINEAGR	Peptide 2.0	Custom order
mAlg8-P2A-mItgb1	IDT	Custom order

CTTCTCTAGGCGCCGGAATTCAGCCACCATGGCAGTG<br>GGCATCACATACACCTGGACCAGGCTGTATGCTTCAGT<br>GTTGACTGGCTCCCTTGTCGGCAGCGGCGCCACAAAC<br>TTCTCTCTGCTAAAGCAAGCAGGTGATGTTGAAGAAAA<br>CCCCGGGCCTGATGACTGCTGGTTCTATTTCACCTATTC<br>AGTGAATGGCTACAATGAAGCTATCGTGCATGTTGTGG<br>AGACTCCAGACTGTCCTTAATACGTAGCTAGCGGATCCCA		
mLama4-P2A-mItgb1<br>CTTCTCTAGGCGCCGGAATTCAGCCACCATGCAGAAAATA<br>TCTTTCTTTGATGGCTTTGAAGTAGGCTTCAATTTCCGAAC<br>ATTACAGCCAAATGGGTTACTATTCTACTACACAGGCAGCG<br>GCGCCACAAACTTCTCTCTGCTAAAGCAAGCAGGTGATGT<br>TGAAGAAAACCCCGGGCCTGATGACTGCTGGTTCTATTTCA<br>CCTATTCAGTGAATGGCTACAATGAAGCTATCGTGCATGTTG<br>TGGAGACTCCAGACTGTCCTTAATACGTAGCTAGCGGATCCC<br>A	IDT	Custom order
**Critical Commercial Assays**		
Fixation/Permeabilization Solution Kit	BD Biosciences	Cat# 555028
Foxp3 / Transcription Factor Staining Buffer Set	eBioscience	Cat# 00-5523-00
Chromium Next GEM Single-cell 5' Reagent Kit v2	10x Genomics	Cat# 100263
Chromium Next GEM Single Cell 5' v2 (Dual Index)	10x Genomics	Cat# CG000330
Qubit HS dsDNA Assay	ThermoFisher	Cat# Q32851
HS DNA Bioanalyzer	Agilent	
**TotalSeq Antibodies**		
CD45 and H-2 MHC class Totalseq™-C0301 anti-mouse Hashtag 1, sequence	BioLegend	Cat# 155861, RRID: AB_2800693
CD45 and H-2 MHC class Totalseq™-C0302 anti-mouse Hashtag 2 Antibody	BioLegend	Cat# 155863, RRID: AB_2800694
CD45 and H-2 MHC class Totalseq™-C0303 anti-mouse Hashtag 3 Antibody	BioLegend	Cat# 155865, RRID: AB_2800695
**Software and algorithms**		
Flow jo_v10.8.1		
GraphPad Prism version 10		
Cell Ranger v.7.1.0	https://support.10xgenomics.com/ single-cell-gene-expression/software	

Seurat R package v.4.3.0.1	https://satijalab.org/seurat/	
scRepertoire v.2.0.0	https://www.borch.dev/uploads/screpertoire/	
ImmGen	https://www.immgen.org	
ggplot2	https://ggplot2.tidy verse.org/index.html	
**Other**		
Fortessa X-20	BD Biosciences	
LSR Fortessa	BD Biosciences	
BD FACSAria II	BD Biosciences	
