# Supplementary material for: Overlapping and Distinct Mechanisms of Effective Neoantigen Cancer Vaccines and Immune Checkpoint Therapy
Source: bioRxiv. 2023 Dec 22:2023.12.20.570816. Preprint. [Version 1] doi: 10.1101/2023.12.20.570816 (PMC10769249; doi:10.1101/2023.12.20.570816)
Supplement: Supplementary file 1 [file NIHPP2023.12.20.570816v1-supplement-2.pdf]

bioRxiv preprint doi: https://doi.org/10.1101/2023.12.20.570816; this version posted December 22, 2023. The copyright holder for this preprint (which was not certified by peer review) is the author/funder, who has granted bioRxiv a license to display the preprint in perpetuity. It is made available under aCC-BY 4.0 International license.

Table S1:

REAGENT or RESOURCE	SOURCE	IDENTIFIER
***In vivo* Antibodies**		
Anti-CTLA-4 (clone 9D9)	Leinco	Cat# C2856, RRID: AB_2829611
Anti-CD279 (PD-1) (clone RMP1-14)	Leinco	Cat# RMP1-14, RRID: AB_2749820
Anti-mouse CD8 (clone YTS 169)	Leinco	Cat# C2442, RRID: AB_2829540
Anti-mouse CD4 (Clone GK1.5)	Leinco	Cat# C2838 RRID: AB_2829596
Mouse IgG2a Isotype.	Leinco	Cat# P381, RRID: AB_2831654
**Flow Antibodies and reagents**		
Anti-CD16/32 (clone 2.4G2)	BD Biosciences	Cat#553141, RRID: AB_394656
Anti-mouse CD45 BV605 (clone 30-F11) (1:800 dilution)	BioLegend	Cat#103140, RRID: AB_2562342
Anti-mouse CD90.2/Thy1.2-PE-Cy7 (clone 30-H12) (1:200 dilution)	BioLegend	Cat#105326, RRID: AB_2201290
Anti-mouse CD8a-BV786 (clone 53–6.7) (1:200 dilution)	BD Bioscience	Cat#563332, RRID: AB_2721167
Anti-mouse CD4-BV711(clone RM4–5) (1:200 dilution)	BioLegend	Cat#100550, RRID: AB_2562099
Anti-mouse CD19-BV650 (clone 1D3) (1:200 dilution)	BD Bioscience	Cat#563235, RRID: AB_2738085
Anti-mouse CD20-BV421 (clone SA275A11) (1:200 dilution)	BioLegend	Cat#150405, RRID: AB_2566540
Anti-mouse CD45R/B220-BUV395 (clone RA3-6B2) (1:200 dilution)	BD Bioscience	Cat# 563793, RRID: AB_2738427
Anti-mouse Nkp46/CD335-FITC (clone 29A1.4) (1:300 dilution)	BioLegend	Cat# 560756, RRID: AB_1727465
Anti-mouse γδ TCR-PE-Cy7 (clone GL3) (1:300 dilution)	BioLegend	Cat# 118124, RRID: AB_11204423
Anti-mouse PD-1-BV421 (clone 29F.1A12) (1:200 dilution)	BioLegend	Cat# 135218, RRID: AB_2561447
Anti-mouse TIM-3 (clone RMT3-23) (1:200 dilution)	BioLegend	Cat# 119727, RRID: AB_2716208
Anti-mouse LAG-3-PerCP-Cy5.5 (clone C9B7W) (1:200 dilution)	BioLegend	Cat# 125212, RRID: AB_2561517
Anti-mouse CD3e-APC (clone 145–2C11) (1:200 dilution),	BioLegend	Cat# 100312, RRID: AB_312677
Anti-mouse CD64-BV421 (clone X54–5/7.1) (1:200 dilution)	BioLegend	Cat# 139309, RRID: AB_2562694
Anti-mouse Ly6G-Alexa Fluor 700 (clone 1A8) (1:400 dilution)	BD Biosciences	Cat# 127622, RRID: AB_10643269
Anti-mouse CX3CR1-FITC (clone SA011F11) (1:1,000 dilution)	BioLegend	Cat# 149020, RRID: AB_2565703
Anti-mouse I-A/I-E-BV650 (clone M5/114.15.2) (1:3,000 dilution)	BD Bioscience	Cat# 563415, RRID: AB_2738192

bioRxiv preprint doi: https://doi.org/10.1101/2023.12.20.570816; this version posted December 22, 2023. The copyright holder for this preprint (which was not certified by peer review) is the author/funder, who has granted bioRxiv a license to display the preprint in perpetuity. It is made available under aCC-BY 4.0 International license.

Anti-mouse CD103-BV421 (clone 2E7) (1:200 dilution)	BioLegend	Cat# 121422, RRID: AB_2562901
Anti-mouse CD24-BV711 (clone M1/69) (1:500 dilution)	BD Bioscience	Cat# 563450, RRID: AB_2738213
Anti-mouse CD11c-BV786 (clone HL3) (1:400 dilution)	BD Biosciences	Cat# 563735, RRID: AB_2738394
Anti-mouse CD11b-APC (clone M1/70) (1:400 dilution)	BioLegend	Cat# 101212, RRID: AB_312795
Anti-mouse F4/80-BUV395 (clone T45–2342) (1:400 dilution)	BD Biosciences	Cat# 565614, RRID: AB_2739304
Anti-mouse CD64-APC (clone X54–5/7.1) (1:400 dilution)	BioLegend	Cat# 139306, RRID: AB_11219391
Anti-mouse CD117-FITC (clone ACK2) (1:100 dilution)	BioLegend	Cat# 135115, RRID: AB_2561633
Anti-mouse CD11b- PerCP-Cy5.5 (clone M1/70) (1:200 dilution)	BioLegend	Cat# 561114, RRID: AB_394002
Anti-mouse PDCA- 1/BST-2 BV650 (clone 927) (1:200 dilution)	BD Biosciences	Cat# 747605, RRID: AB_2744173
Anti-mouse CD172a APC (clone P84) (1:200 dilution)	BioLegend	Cat# 144014, RRID: AB_2564061
Anti-mouse CD274/PDL1-PE (clone MIH5) (1:200 dilution)	BD Biosciences	Cat# 558091, RRID: AB_397018
Anti-mouse FcεRI-PE-Cy7 (clone MAR-1) (1:200 dilution)	BioLegend	Cat# 134326, RRID: AB_2572064
Anti-mouse Mrc1 (CD206)-PE-Cy7 (clone C068C2) (1:400 dilution)	BioLegend	Cat# 141720, RRID: AB_2562248
Anti-mouse FOXP3-FITC (clone FJK-16s)	eBioscience™	Cat# 11-5773-82, RRID: AB_465243
Anti-mouse IFNγ-APC (Clone XMG1.2) (1:200 dilution)	BD Biosciences	Cat# 554413, RRID: AB_398551
Anti-mouse TNF -PE-Cy7 (Clone MP6-XT22) (1:200 dilution)	BD Biosciences	Cat# 561062, RRID: AB_398553
Anti-mouse Granzyme B-PE (Clone NGZB) (1:200 dilution)	eBioscience™	Cat# 12-8898-82, RRID: AB_10870787
Anti-mouse iNOS/Nos2 PE (clone CXNFT) (1:200 dilution)	eBioscience™	Cat# 12-5920-82, RRID: AB_2572642
Zombie Fixable Viability™ Sampler Kit	BioLegend	Cat# 423105
**Chemicals, Peptides, and Gene blocks**		
RPMI-1640	HyClone	Cat# SH30096.02
Trypsin	Genclone	Cat# 25200056
Defined fetal bovine serum	HyClone	Cat# SH30070.03HI
HBSS	Hyclone	Cat# SH30588.02
PBS	Gibco	Cat# 20012027
Sodium Bicarbonate	Gibco	Cat# 25080094

bioRxiv preprint doi: https://doi.org/10.1101/2023.12.20.570816; this version posted December 22, 2023. The copyright holder for this preprint (which was not certified by peer review) is the author/funder, who has granted bioRxiv a license to display the preprint in perpetuity. It is made available under aCC-BY 4.0 International license.

Sodium Pyruvate	Gibco	Cat# 11360070
L-Glutamine	Gibco	Cat# A2916801
ACK lysis buffer	Gibco	Cat# A1049201
Phorbol-12-myristate-13-acetate (PMA)	MilliporeSigma	Cat# 500582
Ionomycin	Fisher	Cat# BP2527-1
Fugene	Promega	Cat# E2311
pMSCV-IRES GFP	addgene	Cat# 20672
Collagenase Type IA	Sigma-Aldrich	Cat# C9891
Poly(I:C) HMW VacciGrade™	InvivoGen	Cat# vac-pic
Gibson Assembly® Cloning Kit	NEB	Cat# E5510S
Mutant Lama4 peptide, sequence VGFNFRTL	Peptide 2.0	Custom order
Mutant Alg8 peptide, sequence ITYTWTRL	Peptide 2.0	Custom order
Mutant Lama4 SLP, sequence<br>QKISFFDGFEVGFNFRTLQPNGLLFYYT	Peptide 2.0	Custom order
Mutant Adpgk SLP, sequence<br>HLELASMTNMELMSSIVHQ	Peptide 2.0	Custom order
Mutant Rpl18 SLP, sequence<br>KAGGKILTFD***R***LALESPK	Peptide 2.0	Custom order
Mutant Dpagt1 SLP, sequence<br>EAGQSLVISASIIVFNL***L***ELEGDYR	Peptide 2.0	Custom order
OVA-I$_{257–264}$ peptide, sequence SIINFEKL	Peptide 2.0	Custom order
Mutant Itgb1 SLP, sequence<br>DDCWFYFTYSVNGY***N***EAIVHVVETPDCP	Peptide 2.0	Custom order
OVA-II$_{323–339}$, sequence<br>ISQAVHAAHAEINEAGR	Peptide 2.0	Custom order
mAlg8-P2A-mItgb1<br>CTTCTCTAGGCGCCGGAATTCAGCCACCATGGCAGTG<br>GGCATCACATACACCTGGACCAGGCTGTATGCTTCAGT<br>GTTGACTGGCTCCCTTGTCGGCAGCGGCGCCACAAAC<br>TTCTCTCTGCTAAAGCAAGCAGGTGATGTTGAAGAAAA<br>CCCCGGGCCTGATGACTGCTGGTTCTATTTCACCTATTC<br>AGTGAATGGCTACAATGAAGCTATCGTGCATGTTGTGG<br>AGACTCCAGACTGTCCTTAATACGTAGCTAGCGGATCCCA	IDT	Custom order
mLama4-P2A-mItgb1<br>CTTCTCTAGGCGCCGGAATTCAGCCACCATGCAGAAAATA<br>TCTTTCTTTGATGGCTTTGAAGTAGGCTTCAATTTCCGAAC<br>ATTACAGCCAAATGGGTTACTATTCTACTACACAGGCAGCG<br>GCGCCACAAACTTCTCTCTGCTAAAGCAAGCAGGTGATGT<br>TGAAGAAAACCCCGGGCCTGATGACTGCTGGTTCTATTTCA<br>CCTATTCAGTGAATGGCTACAATGAAGCTATCGTGCATGTTG	IDT	Custom order

bioRxiv preprint doi: https://doi.org/10.1101/2023.12.20.570816; this version posted December 22, 2023. The copyright holder for this preprint (which was not certified by peer review) is the author/funder, who has granted bioRxiv a license to display the preprint in perpetuity. It is made available under aCC-BY 4.0 International license.

TGGAGACTCCAGACTGTCCTTAATACGTAGCTAGCGGATCCCA		
**Critical Commercial Assays**		
Fixation/Permeabilization Solution Kit	BD Biosciences	Cat# 555028
Foxp3 / Transcription Factor Staining Buffer Set	eBioscience	Cat# 00-5523-00
Chromium Next GEM Single-cell 5 Reagent Kit v2	10x Genomics	Cat# 100263
**TotalSeq Antibodies**		
CD45 and H-2 MHC class Totalseq™-C0301 anti-mouse Hashtag 1, sequence	BioLegend	Cat# 155861, RRID: AB_2800693
CD45 and H-2 MHC class Totalseq™-C0302 anti-mouse Hashtag 2 Antibody	BioLegend	Cat# 155863, RRID: AB_2800694
CD45 and H-2 MHC class Totalseq™-C0303 anti-mouse Hashtag 3 Antibody	BioLegend	Cat# 155865, RRID: AB_2800695
**Software and algorithms**		
Flow jo_v10.8.1		
GraphPad Prism version 10		
**Other**		
Fortessa X-20	BD Biosciences	
LSR Fortessa	BD Biosciences	
BD FACSAria II	BD Biosciences	
