# Supplementary material for: Overlapping and Distinct Mechanisms of Effective Neoantigen Cancer Vaccines and Immune Checkpoint Therapy
Source: bioRxiv. 2023 Dec 22:2023.12.20.570816. Preprint. [Version 1] doi: 10.1101/2023.12.20.570816 (PMC10769249; doi:10.1101/2023.12.20.570816)

Supplemental Figure 1

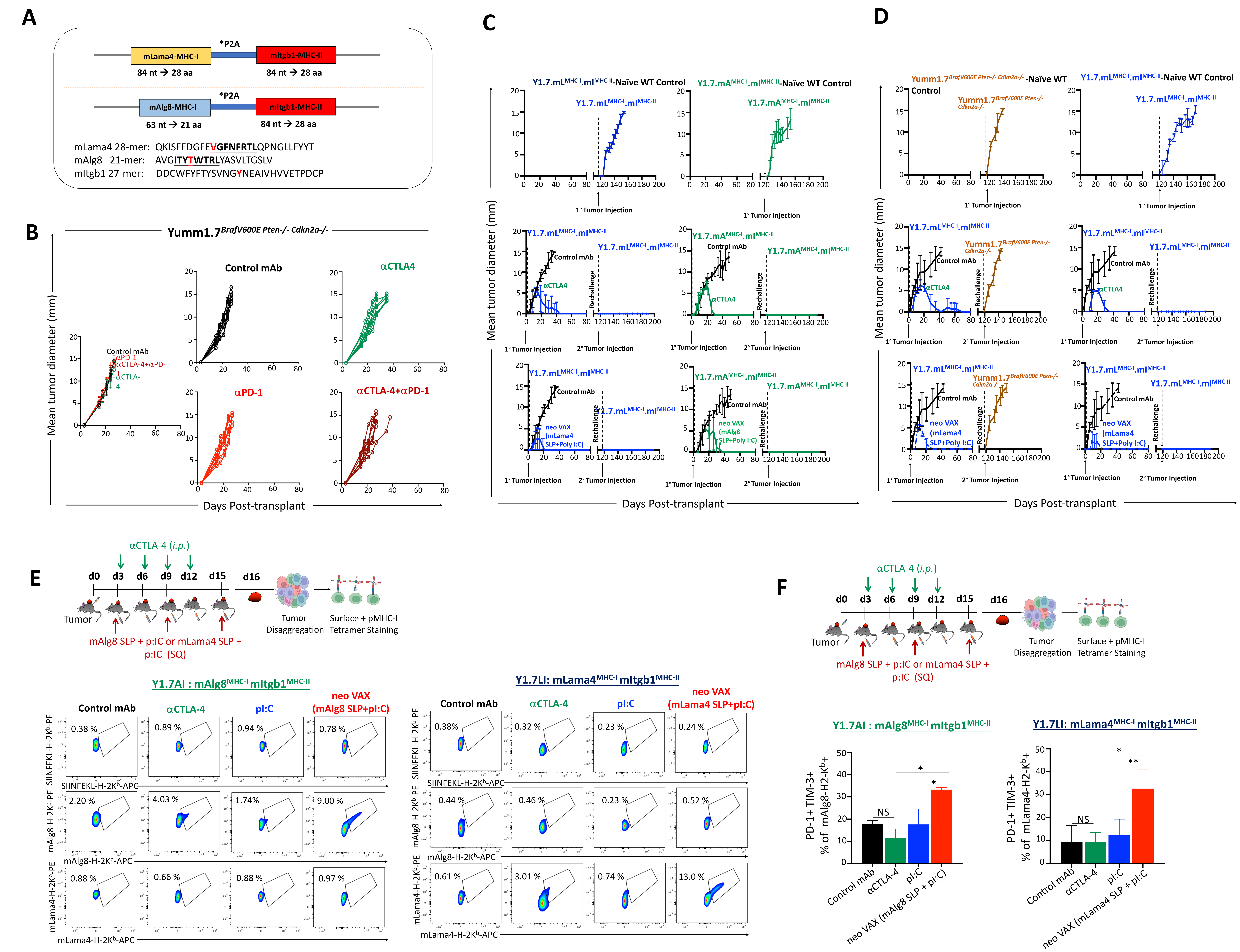

Supplemental Figure 2

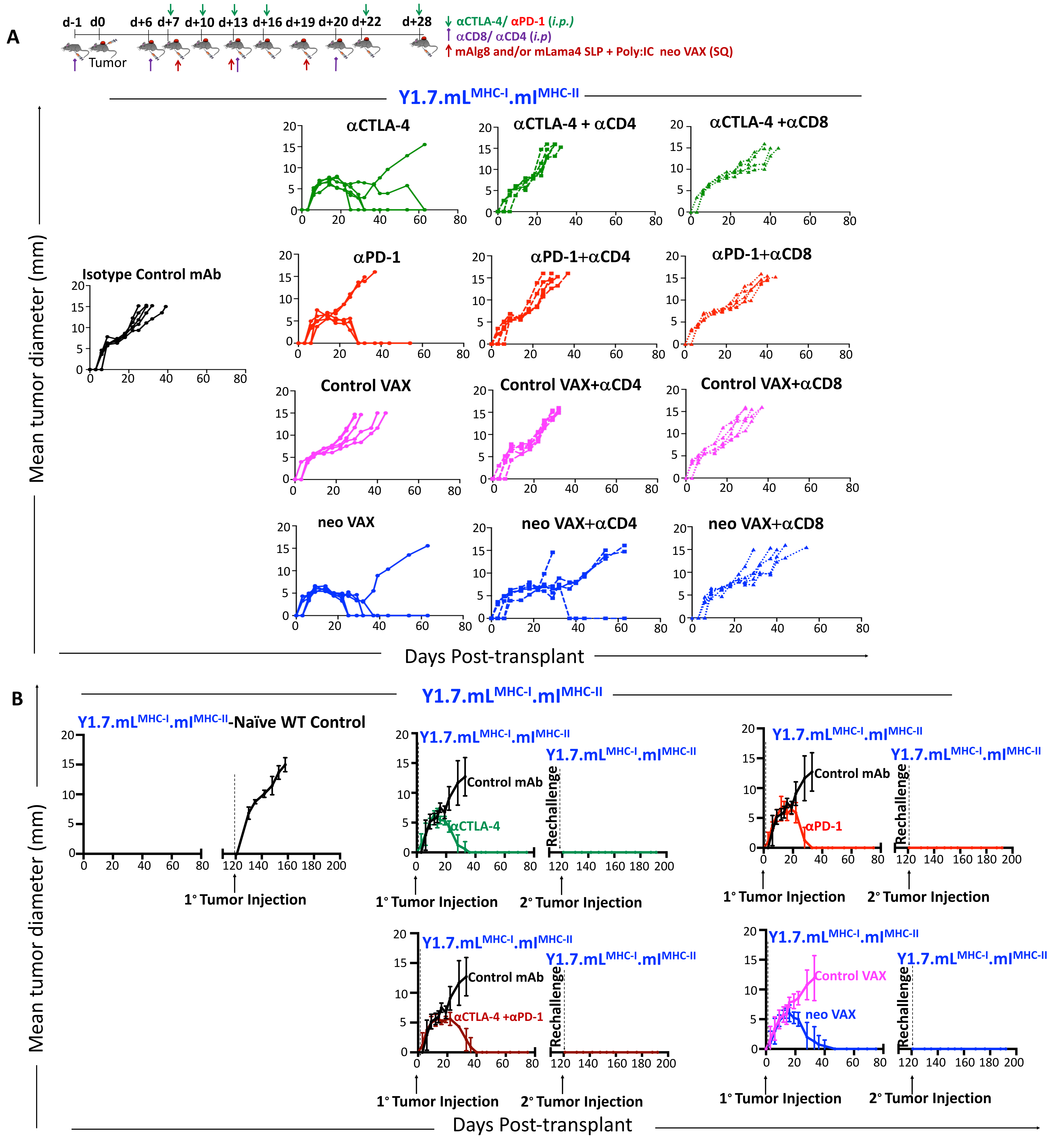

Supplemental Figure 3

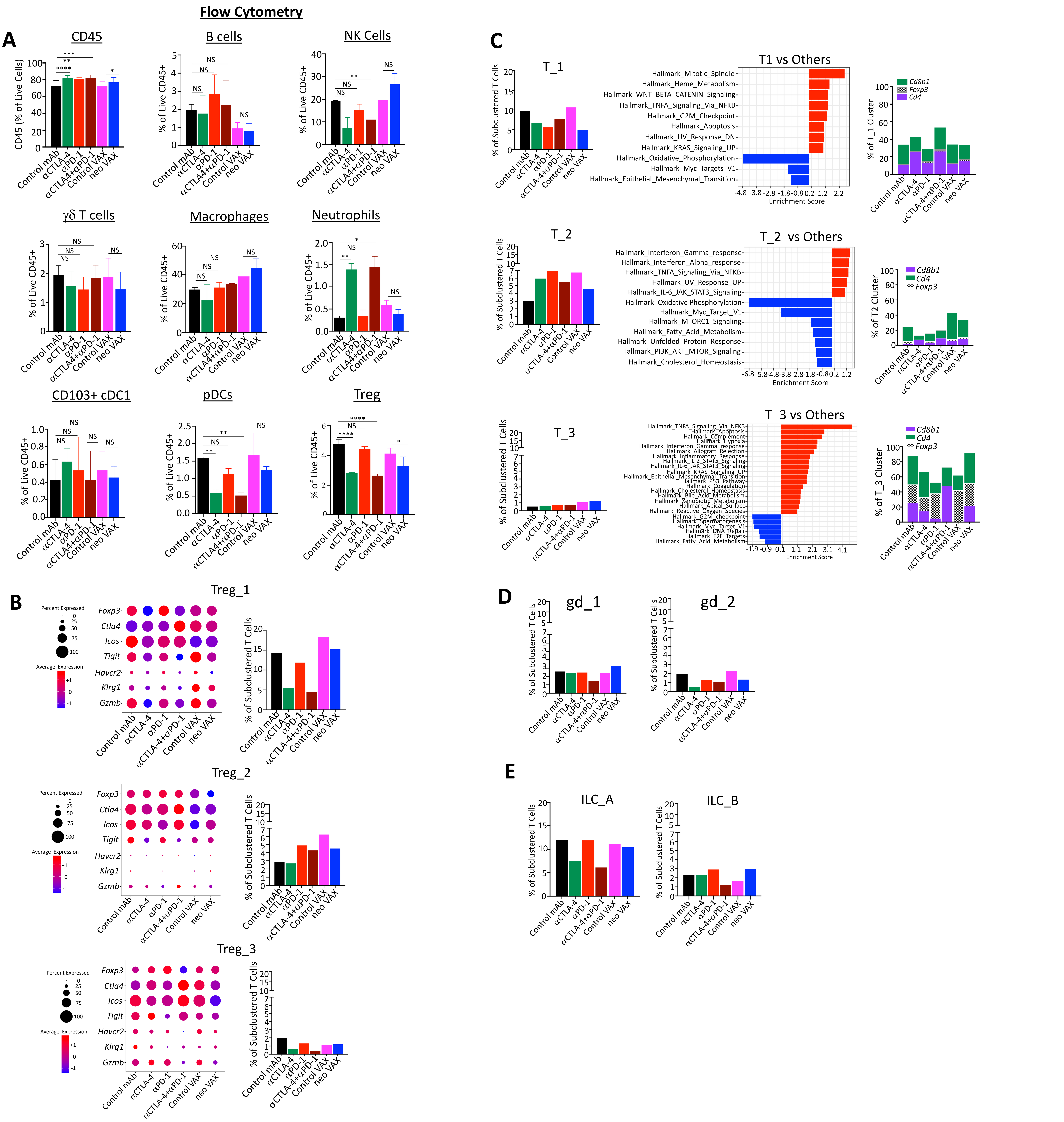

Supplemental Figure 4

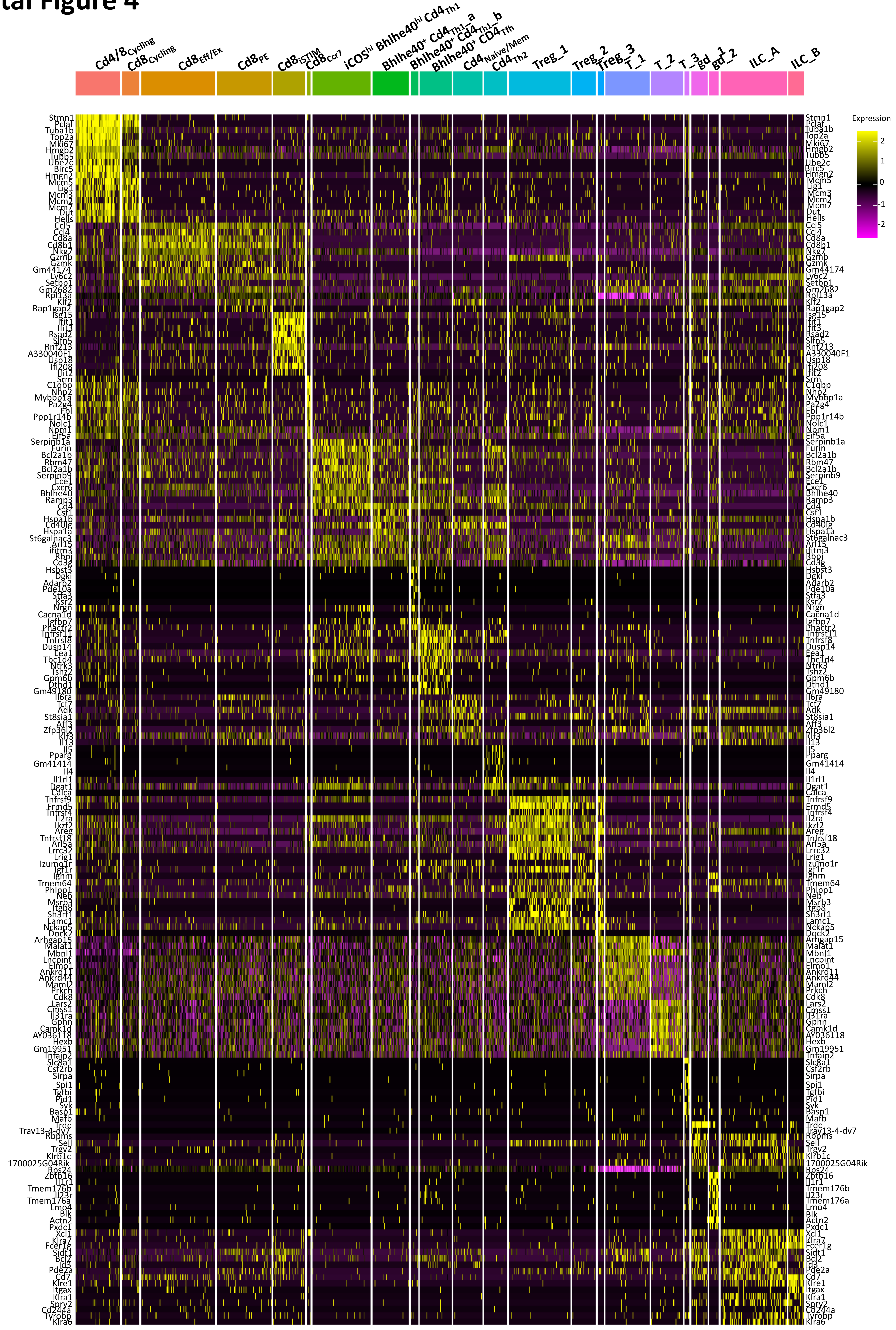

Supplemental Figure 5

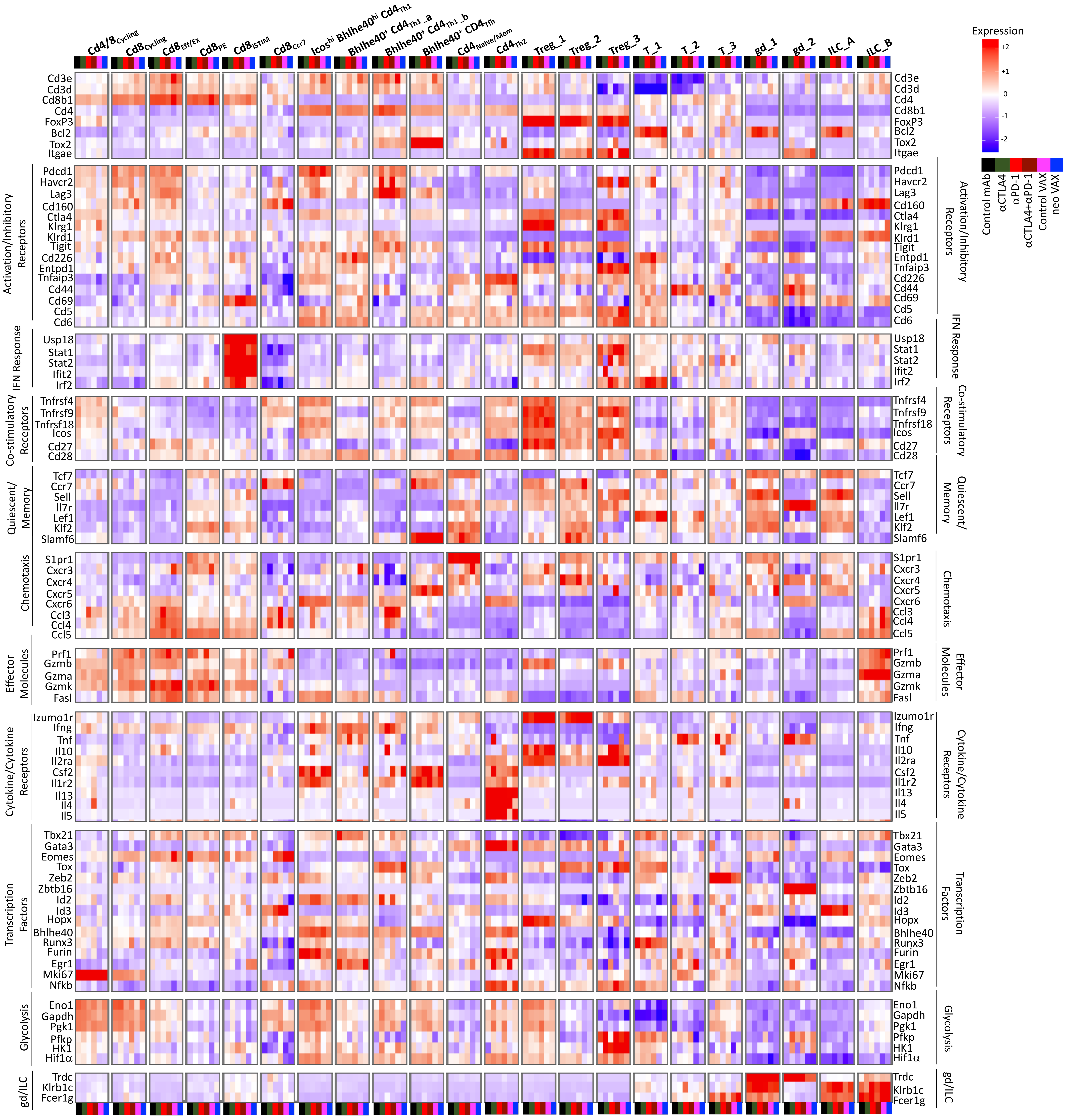

Supplemental Figure 6

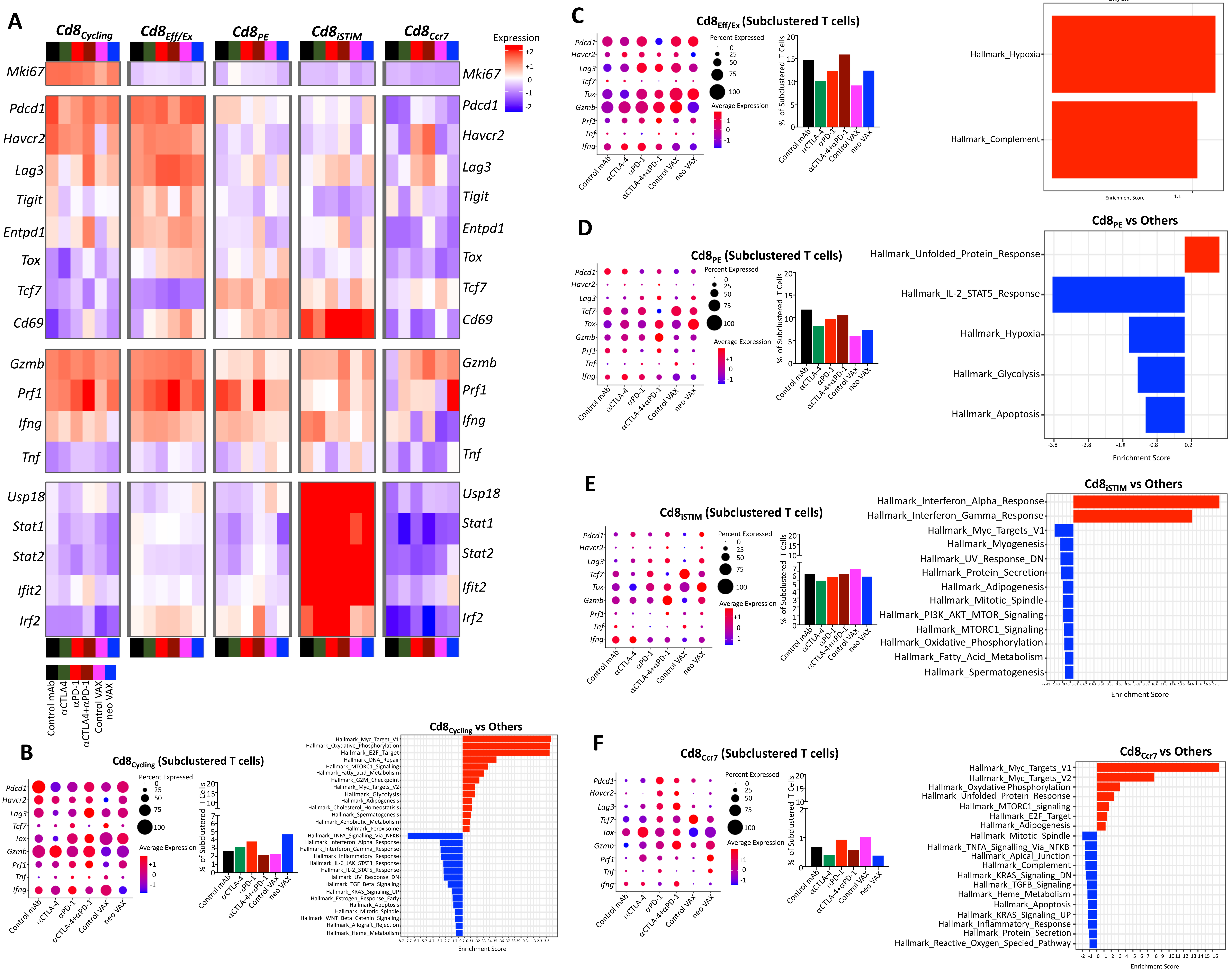

Supplemental Figure 7

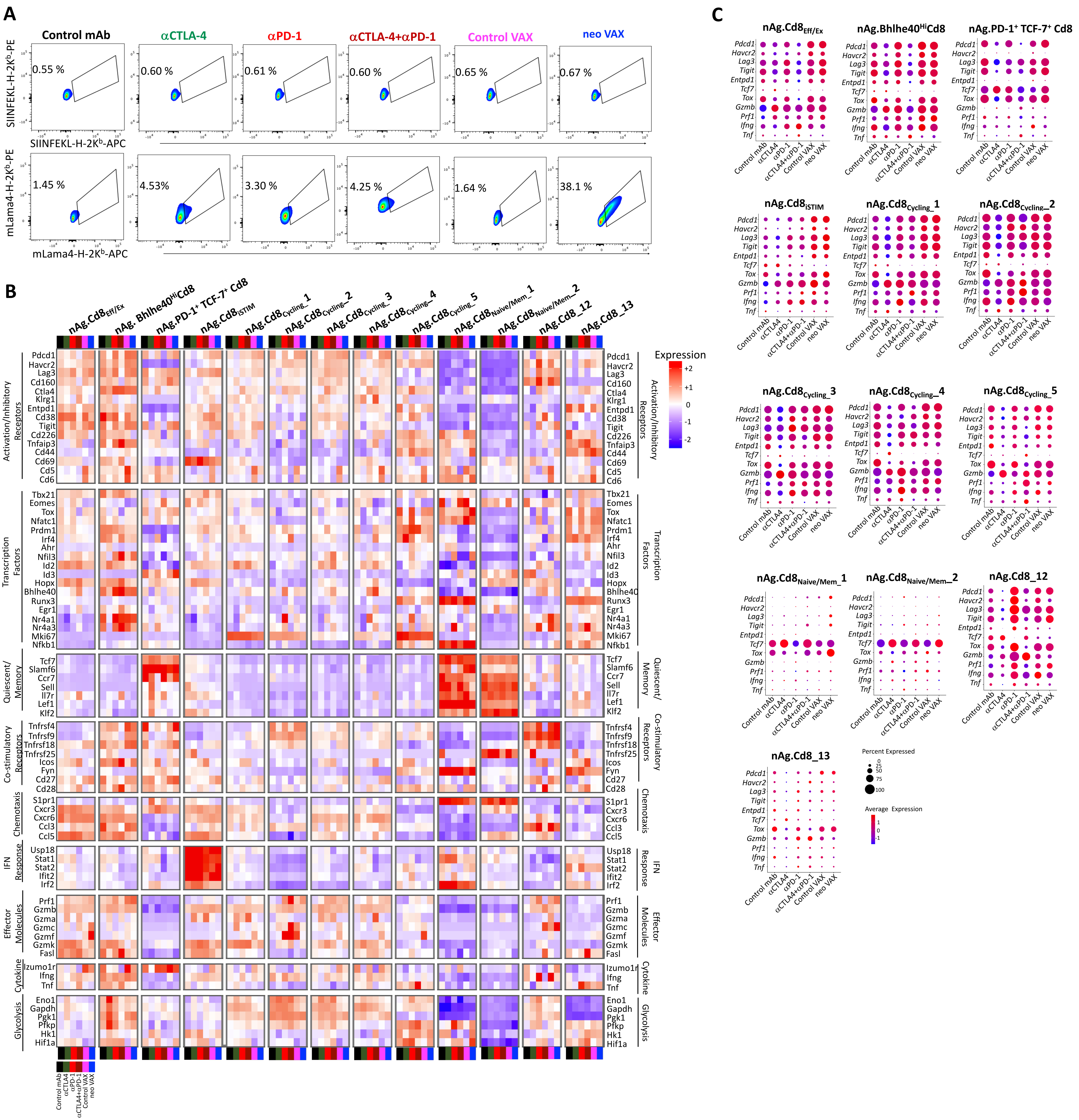

Supplemental Figure 8

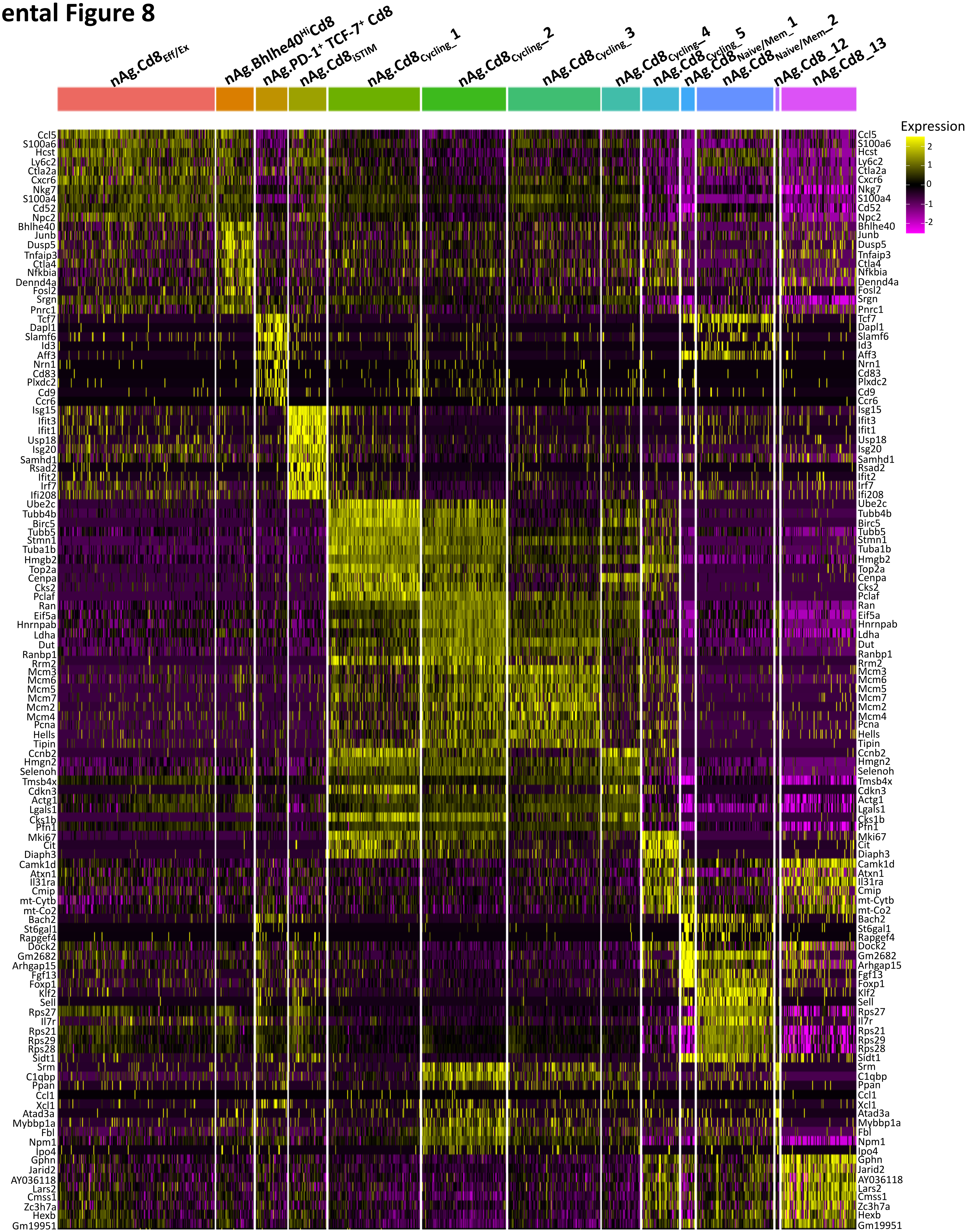

Supplemental Figure 9

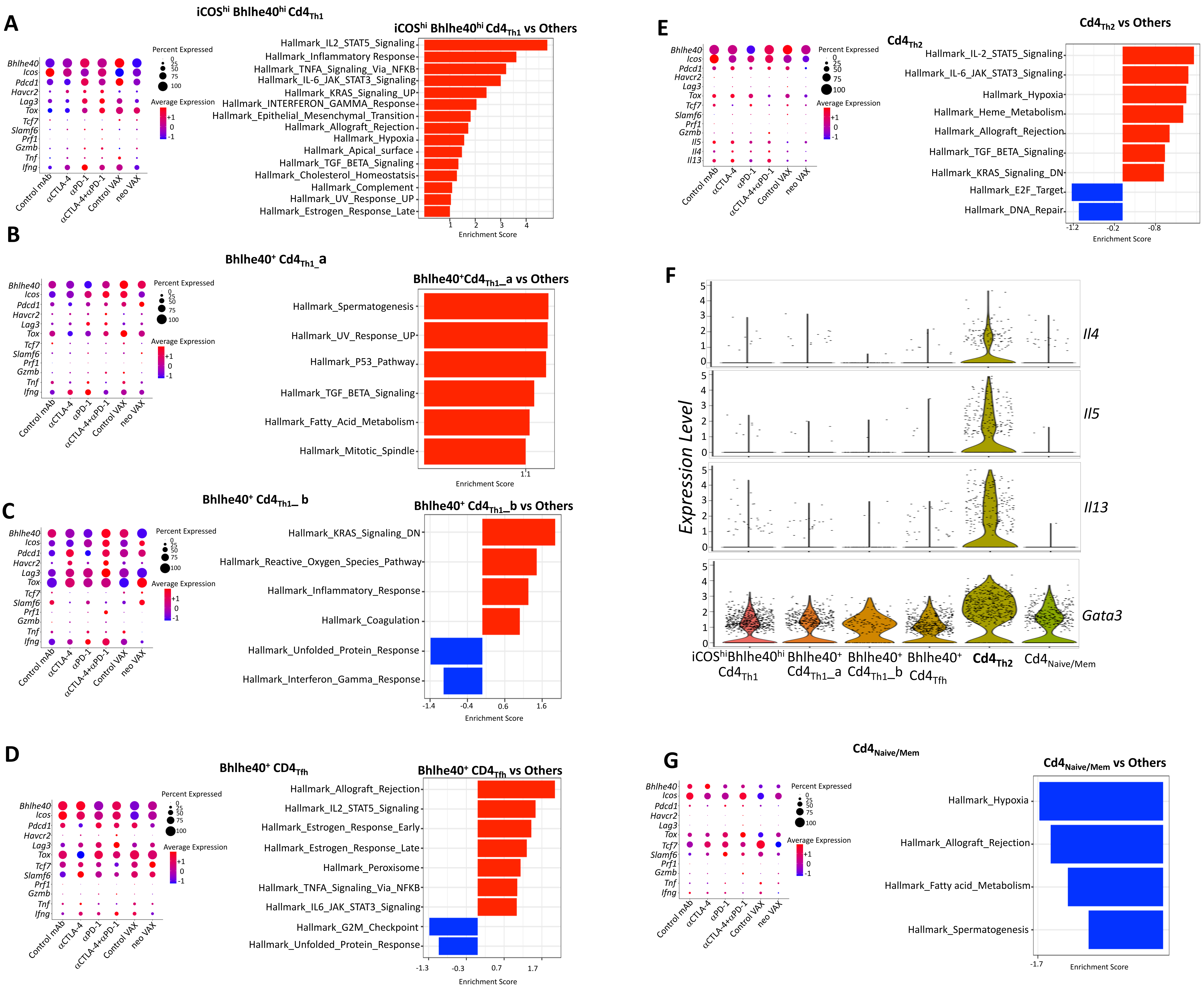

Supplemental Figure 10

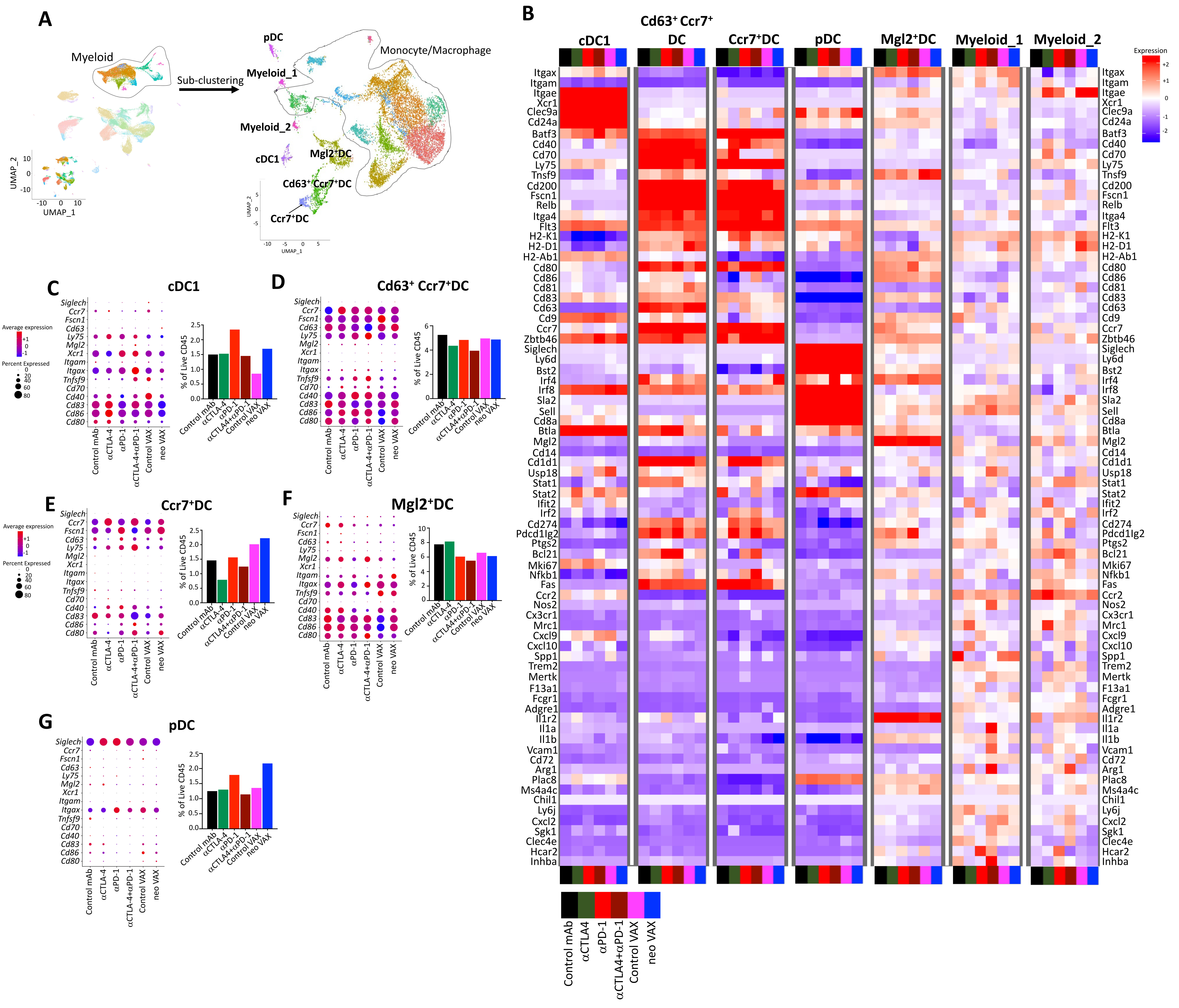

Supplemental Figure 11

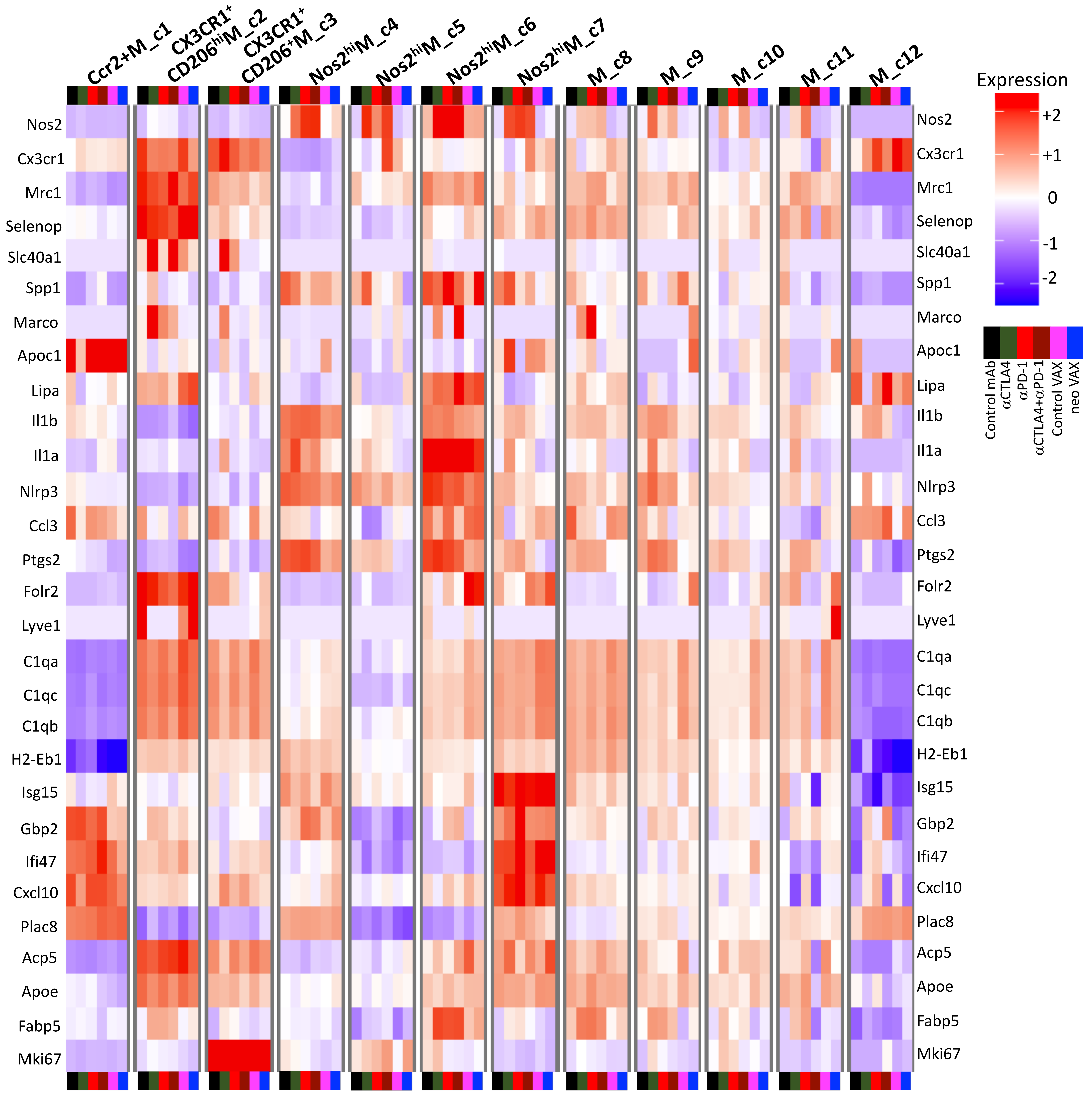

Supplemental Figure 12

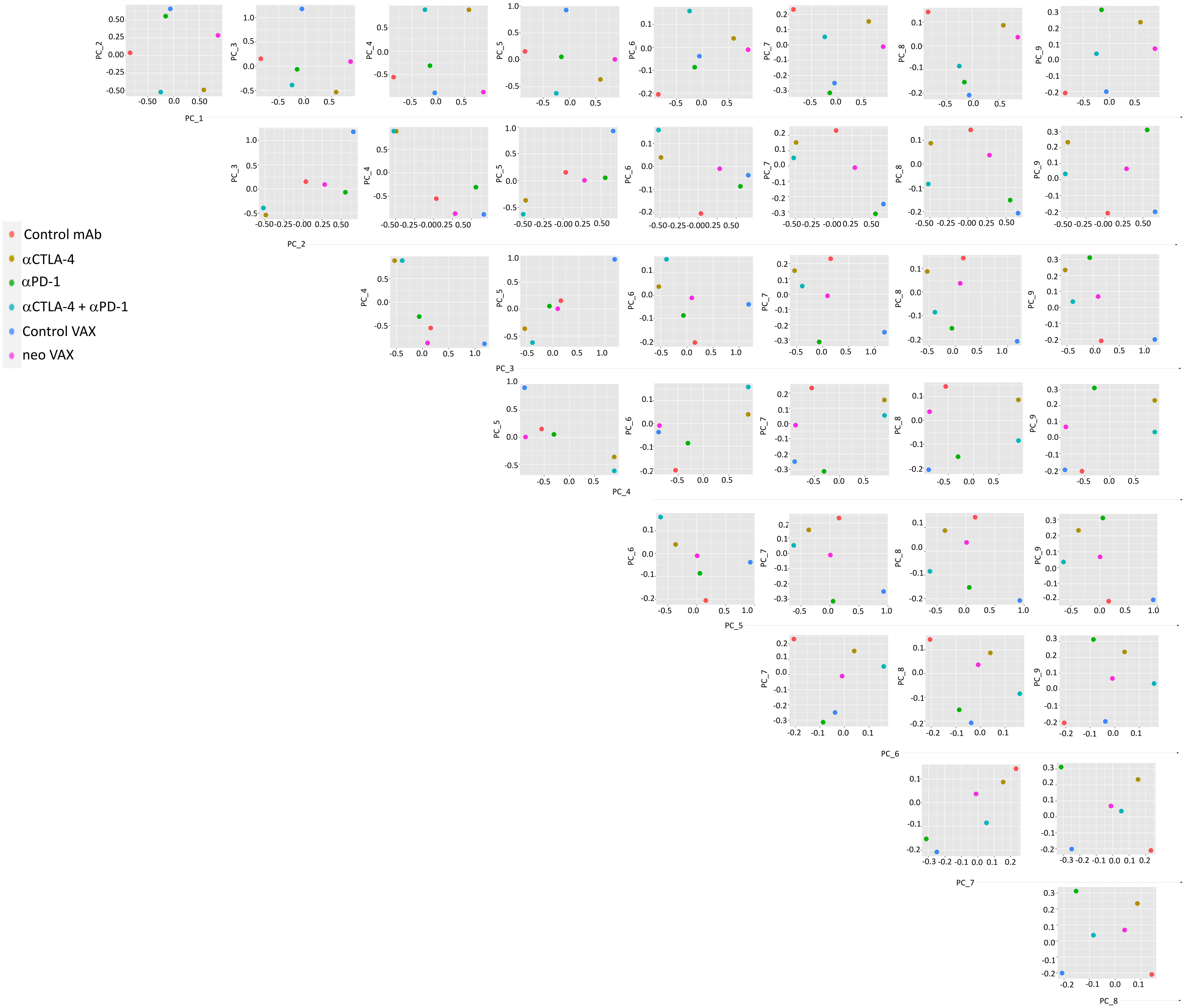

Supplemental Figure 13

Myeloid Subsets

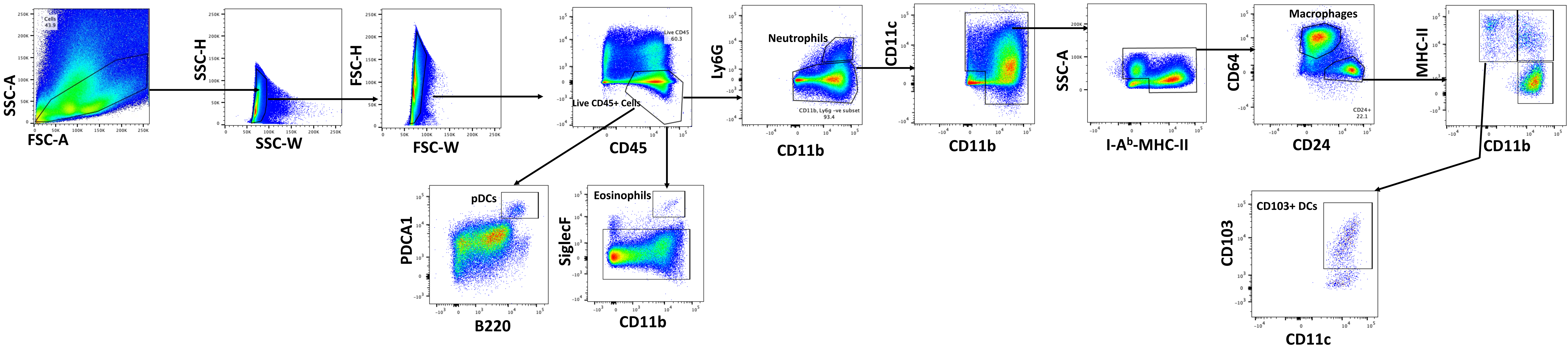

Lymphoid Subsets

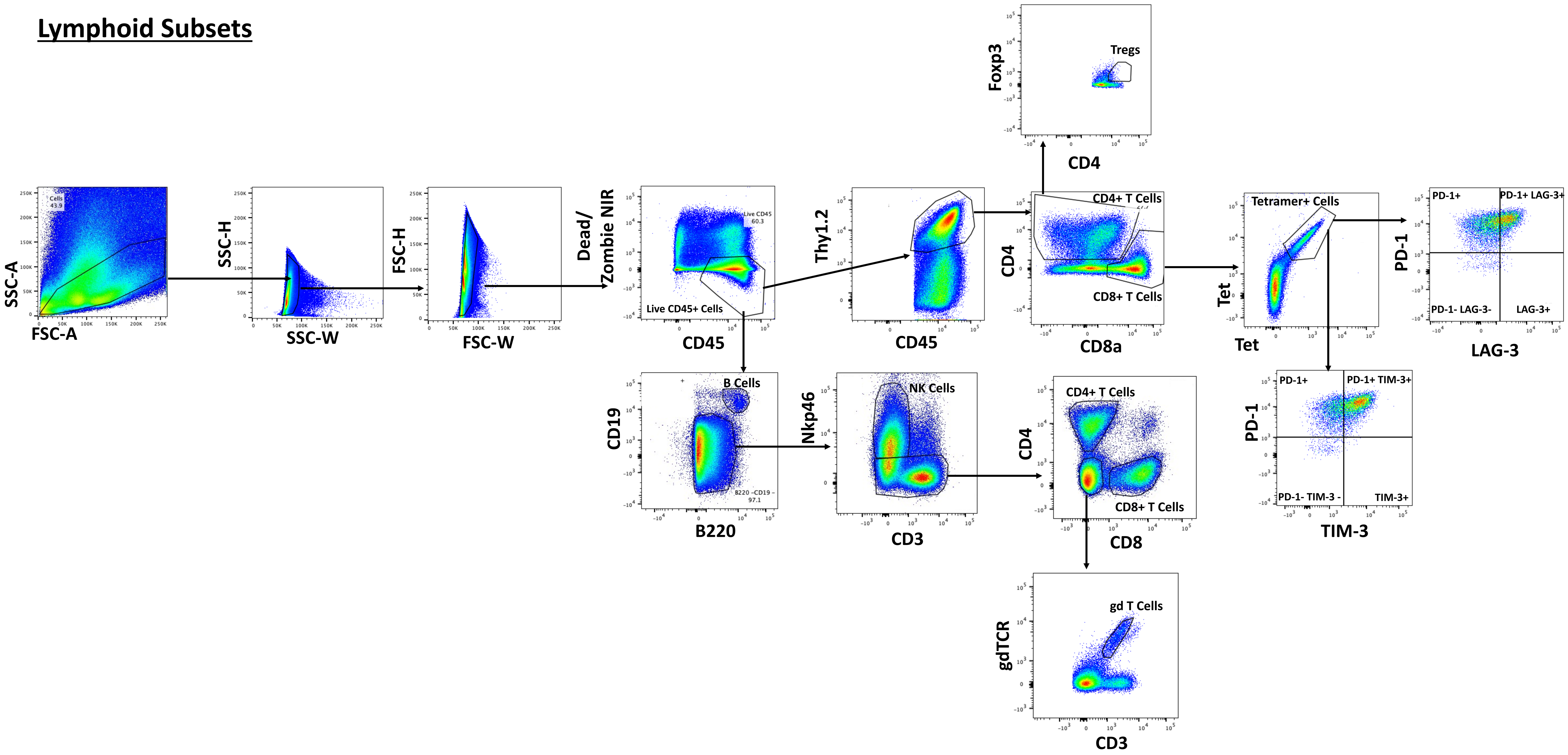

Supplement: Supplementary file 2 [file media-1.pdf]
